# In silico prediction of potential inhibitors for SARS-CoV-2 Omicron variant using molecular docking and dynamics simulation-based drug repurposing

**DOI:** 10.1007/s00894-023-05457-z

**Published:** 2023-02-20

**Authors:** Eslam A. R. Mohamed, Islam M. Abdel-Rahman, Magdi E. A. Zaki, Ahmad Al-Khdhairawi, Mahmoud M. Abdelhamid, Ahmad M. Alqaisi, Lyana binti Abd Rahim, Bilal Abu-Hussein, Azza A. K. El-Sheikh, Sayed F. Abdelwahab, Heba Ali Hassan

**Affiliations:** 1 Department of Chemistry, Faculty of Science, Minia University, Minia, 61511 Egypt; 2 Department of Pharmaceutical Chemistry, Faculty of Pharmacy, Deraya University, New-Minia, 61519 Minia Egypt; 3 Department of Chemistry, Faculty of Science, Imam Mohammad Ibn Saud Islamic University (IMSIU), Riyadh, Saudi Arabia; 4 Department of Biological Science and Biotechnology, Faculty of Science and Technology, Universiti Kebangsaan Malaysia, 43600 Bangi, Selangor Malaysia; 5 Department of Pharmaceutical Chemistry, Faculty of Pharmacy, Al-Azhar University, Asyut, 71524 Egypt; 6 Chemistry Department, University of Jordan, Amman, 11942 Jordan; 7 Present Address: School for Engineering of Matter, Transport and Energy, Arizona State University, Tempe, AZ 85287 USA; 8 Department of Medicine, Hospital Tuanku Ampuan Najihah, Kuala Pilah, Negeri Sembilan Malaysia; 9 Albayader Specialty Hospital, Amman, Jordan; 10 Present Address: Department of General Surgery, Cumberland Infirmary Hospital, Carlisle, England; 11 Basic Health Sciences Department, College of Medicine, Princess Nourah bint Abdulrahman University, P.O. 13 Box 84428, Riyadh, 11671 Saudi Arabia; 12 Department of Pharmaceutics and Industrial Pharmacy, College of Pharmacy, Taif University, PO Box 11099, Taif, 21944 Saudi Arabia; 13 Department of Pharmacognosy, Faculty of Pharmacy, Sohag University, Sohag, 82524 Egypt

**Keywords:** B.1.1.529, COVID-19, Drug score, Molecular docking, Molecular dynamics, Omicron

## Abstract

**Background:**

In November 2021, variant B.1.1.529 of severe acute respiratory syndrome coronavirus 2 (SARS-CoV-2) was identified by the World Health Organization (WHO) and designated Omicron. Omicron is characterized by a high number of mutations, thirty-two in total, making it more transmissible than the original virus. More than half of those mutations were found in the receptor-binding domain (RBD) that directly interacts with human angiotensin-converting enzyme 2 (ACE2). This study aimed to discover potent drugs against Omicron, which were previously repurposed for coronavirus disease 2019 (COVID-19). All repurposed anti-COVID-19 drugs were compiled from previous studies and tested against the RBD of SARS-CoV-2 Omicron.

**Methods:**

As a preliminary step, a molecular docking study was performed to investigate the potency of seventy-one compounds from four classes of inhibitors. The molecular characteristics of the best-performing five compounds were predicted by estimating the drug-likeness and drug score. Molecular dynamics simulations (MD) over 100 ns were performed to inspect the relative stability of the best compound within the Omicron receptor-binding site.

**Results:**

The current findings point out the crucial roles of Q493R, G496S, Q498R, N501Y, and Y505H in the RBD region of SARS-CoV-2 Omicron. Raltegravir, hesperidin, pyronaridine, and difloxacin achieved the highest drug scores compared with the other compounds in the four classes, with values of 81%, 57%, 18%, and 71%, respectively. The calculated results showed that raltegravir and hesperidin had high binding affinities and stabilities to Omicron with Δ*G*_binding_ of − 75.7304 ± 0.98324 and − 42.693536 ± 0.979056 kJ/mol, respectively. Further clinical studies should be performed for the two best compounds from this study.

**Graphical Abstract:**

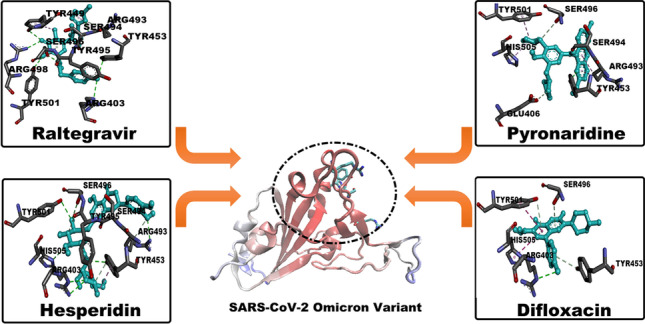

**Supplementary Information:**

The online version contains supplementary material available at 10.1007/s00894-023-05457-z.

## Introduction

As established previously, the emergence of coronavirus disease in 2019 (COVID-19) has been attributed to severe acute respiratory syndrome coronavirus 2 (SARS-CoV-2) [1–3]. Since its discovery in December 2019, SARS-CoV-2 has been undergoing a series of mutations that have resulted in increased transmissibility and notable resistance [4, 5]. Consequently, many virus lineages were declared variants of concern (VOCs) by the World Health Organization (WHO). The reported VOCs first began in the UK as B.1.1.7 (alpha). Then, B.1.351 (beta) was reported in South Africa. The third, P.1 (Gamma) has appeared in Brazil. Recently, B.1.617.2 (Delta) and B.1.1.529 (Omicron) were recognized in India and South Africa, respectively.

SARS-CoV-2 Omicron was first discovered in late November 2021 as a multi-mutagenic virus that is rapidly spreading worldwide. Newly approved vaccines showed no effect on Omicron [6, 7]. What is also distinguishable in Omicron is that thirty amino acids were substituted in the spike protein (S-protein) as compared to the SARS-CoV-2 wild type. Fifteen amino acid substitutions out of them are located in the receptor-binding domain (RBD). The main crucial function of RBD is its ability to bind to the human angiotensin-converting enzyme 2 (hACE2) receptor, which exists on the surface cells of the throat and the epithelial cells of the lung [8, 9]. This binding results in a fusion between the S-protein and the cell membrane of human cells, which causes replication of the genetic materials within the cells of the host [10]. Studies associated with the in vitro protocol suggest that two mutations, namely Q498R and N501Y, are the main cause of the increased binding affinity in the RBD-hACE2 complex [11].

**Fig. 1:**
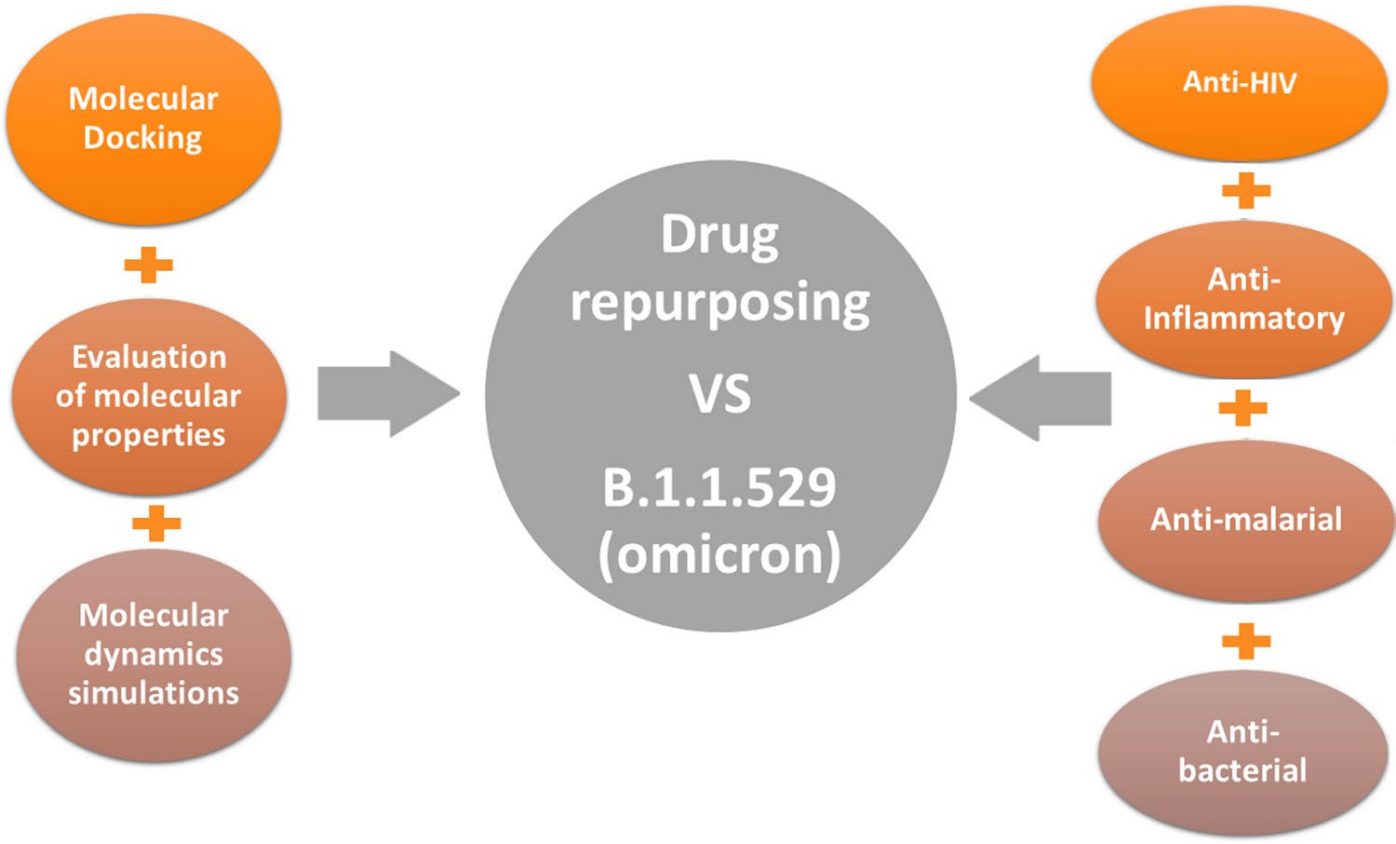
Schematic representation of the overall workflow applied in the current study

In the current situation, there is an urgent need for potential anti-viral agents to stop the spread of the virus. To achieve this goal, the approach of “drug repurposing” was chosen as a rapid way to inhibit Omicron activity. The key features of the repurposing approach are time saving, cost saving, and efficacy, as well as the use of FDA-approved drugs [12, 13]. The current study inspected four classes containing previously used anti-COVID-19 drugs using various in silico methods. Figure 1 shows a schematic illustration of the in silico approaches used in the filtration process and the selected classes of inhibitors.

Mefloquine [14], artemisinin [15], chloroquine, and hydroxychloroquine [16] are the main anti-malarial drugs that were previously tested as COVID-19 inhibitors [17–26]. In the class of anti-inflammatory drugs, dexamethasone [27, 28], hesperidin [29], diosmin [30], and colchicine [31] were recorded as potential COVID-19 inhibitors. In respect of anti-HIV drugs [32–47], darunavir [48], raltegravir, indinavir, and etravirine proved their efficacy [40]. Through searching in previous studies, antibiotics, specifically fluoroquinolones, were used to counter COVID-19 infection [49–57]. Levofloxacin, moxifloxacin, and ciprofloxacin were the most commonly utilized anti-COVID-19 antibiotics [58, 59].

The main objective of this study is to identify the best drug from each class to inhibit the activity of Omicron and limit its spread. As a first step, a molecular docking study was performed to determine the potential activity of all inhibitors. Based on the estimated docking scores, the molecular properties of the five highest-ranked inhibitors were evaluated. Using the drug-likeness values and drug scores, molecular dynamics simulations (MD) were performed over 100 ns, followed by binding energy calculations using a molecular mechanics-generalized Born surface area (MM/GBSA) approach implemented for the best inhibitor from each class. The results obtained in this project suggest that the identified compounds could be used to inhibit the activity of the newly emerged Omicron variant of SARS-CoV-2, which should be studied in vitro and in vivo.

## Computational methodology

### Protein selection and preparation

In view of the molecular docking study, the recently deposited 3D-crystal structure of the receptor-binding domain of Omicron (O-RBD) in a protein data bank (PDB) was chosen and subsequently prepared (PDB ID: 7QNW, 2.40 Å). Assigning the protonation state of 7QNW amino acids was done using the H +  + server (http://biophysics.cs.vt.edu/H + +). In addition, all missed hydrogen atoms were added [60, 61]. To investigate pKa for residues, some parameters were adjusted in the H +  + server such as external dielectric, internal dielectric, salinity, and pH to be equaled to 80, 10, 0.15, and 6.5, respectively.

### Ligand preparation

In the present study, seventy-one drugs from the four major anti-viral classes were examined to determine the likely interaction of each category against O-RBD. The structures of the tested compounds were obtained from the PubChem database (https://pubchem.ncbi.nlm.nih.gov) and stored in Spatial Data File (SDF) format. In addition, molecular minimization using the MM2 force field was performed using Chem3D Pro 12.0 software.

### Molecular docking analysis

The main purpose of molecular docking analysis is to assign the binding free energy of the studied compounds and give an indication of to what extent the protein is inhibited through a defined value, which is called docking score. AutoDock Vina [62] is a computational program, which estimates the docking score of compounds in an accurate manner by evaluating nine binding modes of a ligand inside the protein active site. Based on the AutoDock protocol, the SARS-CoV-2 Omicron structure was converted into pdbqt format using MGTools 1.5.6 [63]. Except for the exhaustiveness parameter, which was set to 200, all molecular docking parameters were left at their default levels. A grid box with XYZ dimensions of 25 × 25 × 25 (Å) and a spacing value of 1.00 Å was used to comprise the mutated residues of the RBD. The grid center was located at − 24.35, 21.29, and − 30.735 (XYZ coordinates) for SARS-CoV-2 Omicron. All nine poses were explored and only the highest one of binding energy was considered for further investigations. Different interactions of protein–ligand complexes were inspected through BIOVIA Discovery Studio [64].

### In silico evaluation of molecular properties

To determine molecular properties that are should present in drug candidates, the Osiris property explorer was used. In order to evaluate how the discovered compounds are risky, properties related to irritation, mutagenicity, tumorigenicity, and reproductive effects were tested. Furthermore, additional parameters like clogP (n-octanol–water partition coefficient), MT (molecular weight), and logS (aqueous solubility) were studied. Before implementing the molecular dynamics (MD) simulations, all of that parameters were blended to generate one unique result called drug score (DS), which shows the total drug potential. Estimation of the DS can be executed by applying the following equation:

$$DS=\prod \left(\frac{1}{2}+\frac{1}{2}{S}_{i}\right). \prod {t}_{i}$$

where (*S*_*i*_) is:

$${S}_{i}=\frac{1}{1+{e}^{a{p}_{i}+b}}$$

*Si* symbolizes the contributions calculated instantly from cLogP (*c*_octanol_/*c*_water_), molecular weight (Mwt), and drug-likeness (pi) via the second equation which represents a spline curve. Parameters *a* and *b* are (1, 5) for logS, (1, − 5) for cLogP, (0.012, − 6) for MT, and (1, 0) for drug-likeness. *t*_*i*_ expresses the contributions estimated from the four main types of toxicity risk (tumorigenic, mutagenic, irritant, and reproductive).

### Molecular dynamics simulations

The system was prepared using the CHARMM-GUI [65–67] interface, which was supported by the CHARMM36 force field [68]. The NAMD 2.13 software [69] was used for operating all simulations. Dimensions of the periodic boundary conditions were set at 115.2 Å^3^. TIP3P explicit water solvation model [70] was employed. The CHARMM General Force Field (CGenFF) [71] was used to produce the parameters for the investigated systems. Each system was then neutralized with a sufficient number of Na + /Cl- ions. Minimization, heating, equilibration, and production steps were included in the MD protocols. All MD simulations were performed using a 2 fs time step of integration. The equilibration and production were carried out in the canonical (NVT) ensemble and the isothermal–isobaric (NPT) ensemble, respectively. Using the Nosé–Hoover–Langevin piston barostat [72], with a Langevin piston decay of 0.05 ps and a period of 0.1 ps, the pressure was set at 1 atm for the 100 ns of MD production. The Langevin thermostat [73] was used to set the temperature at 298.15 K. A distance cutoff of 12.0 Å along with a pair list distance of 16 Å was applied to short-range non-bonded interactions. The Lennard–Jones interactions were evaluated with an 8.0 Å cutoff. The particle-mesh Ewald (PME) method [74, 75] was employed to treat long-range electrostatic interactions, with a grid spacing of 1.0 for all simulation cells. The SHAKE algorithm was used to restrict all covalent bonds containing hydrogen atoms. The same procedure was applied for all MD simulations to ensure uniformity.

### Binding energy calculations

For the relative binding energy estimates, the molecular mechanics-generalized Born surface area (MM/GBSA) procedure executed in the MOLAICAL code was used. This can be described by using the following equations,

$${\Delta G}_{binding}= {\Delta G}_{C}-{\Delta G}_{P}- {\Delta G}_{L}$$

$${\Delta G}_{binding}=\Delta H-T\Delta S= {\Delta E}_{MM}+{\Delta G}_{Sol}- T\Delta S$$

where Δ*G*_*C*_, Δ*G*_*P*_, and Δ*G*_*L*_ represent the binding energy value of the complex, protein, and ligand, respectively. As well, Δ*E*_*MM*_, Δ*G*_*Sol*_, and -*T*Δ*S* represent the gas phase molecular mechanics change, the solvation Gibbs energy, and the conformational entropy, respectively. The term Δ*E*_*MM*_ is equal to the total of changes in electrostatic energies added to the van der Waals energies and the internal energies. The term Δ*G*_Sol_ is the sum of both the polar and nonpolar solvation. The normal mode of analysis was used to compute -*T*∆*S*. For the MM/GBSA computations, the solvent dielectric constant was set to 78.5 and the surface tension constant was set to 0.03012 kJ mol^−1^Å^2^.

## Results and discussion

The S- protein of SARS-CoV-2 plays a pioneering role in facilitating virus entry and fusion with the cell membrane. This is attributed to its binding to the hACE2 receptor. In recent months, a multimutated virus, the SARS-CoV-2 Omicron variant, has emerged that exhibits significantly increased infectivity. These variants are located in a specific region of residues called RBD, which ranges from 319 to 541 [76].

### Molecular docking analysis

To explore the binding affinity of the studied compounds against the RBD of Omicron (O-RBD), an effective, accurate, and rapid computational approach called molecular docking was employed. Different types of interactions between ligand atoms and protein residues identify the extent of inhibition of the target protein. Hydrogen bonding (HB) is considered the most important of these interactions as it provides noticeable protein–ligand stability. Moreover, other types of interactions, such as pi-alkyl, pi-cation, and pi-sigma, were also observed. The molecular docking of four anti-viral classes against O-RBD, as well as binding features between each ligand and the mutated residues, was discussed and explained below.

### Anti-inflammatory drugs

**Fig. 2:**
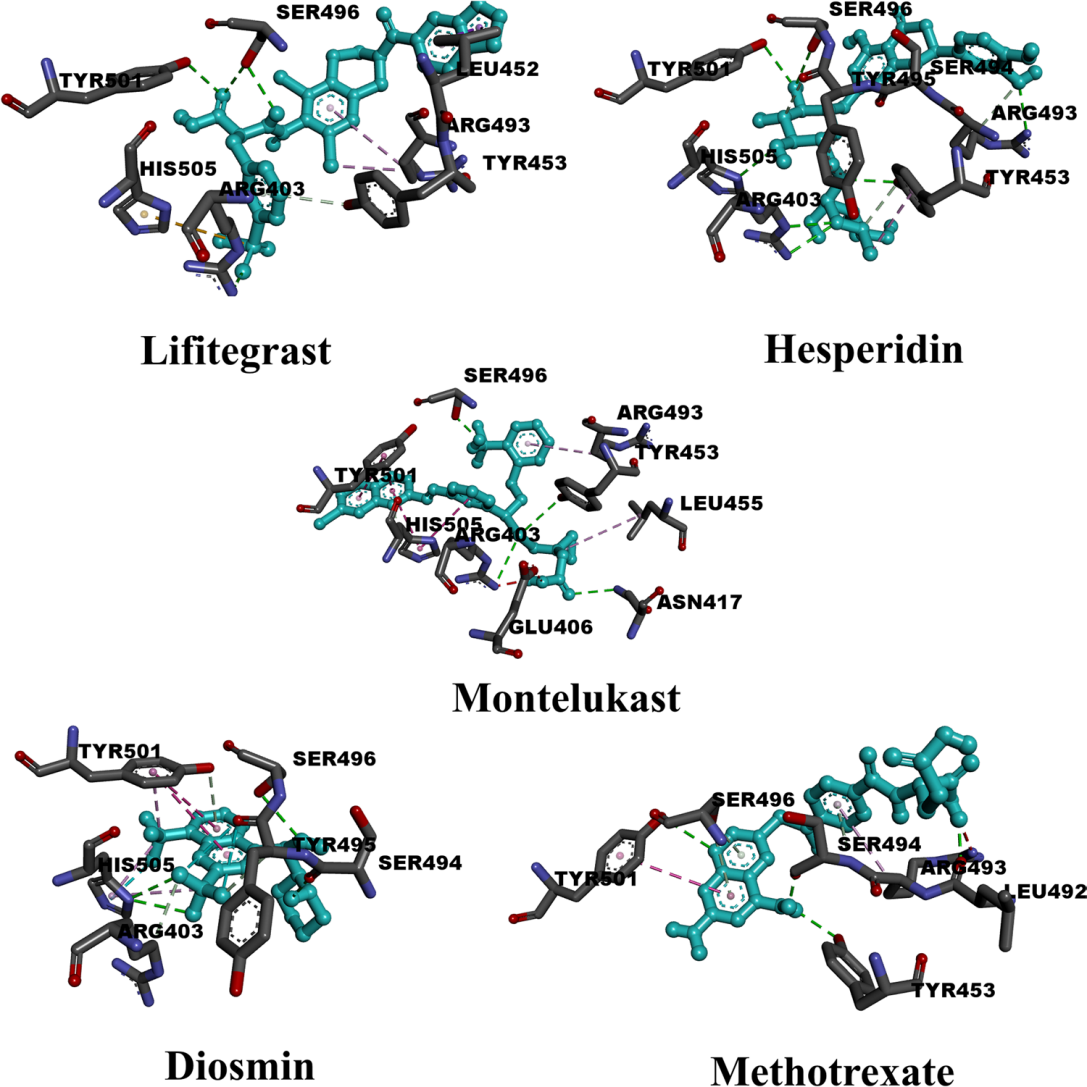
3D representations of binding modes of the best five anti-inflammatory drugs based on the estimated values of docking score

Fifteen anti-inflammatory drugs were inspected using the molecular docking technique. Calculated results (Table S1) revealed that four compounds achieved docking scores below − 6.0 kcal/mol, whereas five compounds achieved docking scores above − 7.0 kcal/mol. The highest value (− 8.10 kcal/mol) was scored by lifitegrast, whereas colchicine exhibited low inhibition with a value of − 5.30 kcal/mol. The average value of molecular docking scores for all inspected anti-inflammatory drugs was estimated to be equal to − 6.51 kcal/mol. Table S1 demonstrates the binding features of this class of inhibitors against the Omicron variant of SARS-CoV-2. Table S2 also includes the dataset of those compounds. Figure 2 depicts the full set of interactions for the top five compounds. All drugs formed more than one conventional hydrogen bond with active site residues. In detail, dexamethasone formed two hydrogen bonds with ASN417 (2.91 Å) and HIS505 (3.47 Å). Initially, for conventional hydrogen bonds, lifitegrast formed two bonds with SER496 (2.90, 2.98 Å) and a single one with ARG403 (2.89 Å) and TYR501 (2.90 Å). As well, only one carbon-hydrogen bond with TYR453 (4.05 Å) can be noticed. Regarding the pi-alkyl type of interaction, lifitegrast formed three bonds, two of them with ARG493 (3.88, 5.27 Å) and the other with LEU452 (5.00 Å). It also formed a pi-sigma interaction with LEU452 (3.89 Å). As lifitegrast contains a sulfonyl group in its structure, a pi-sulfur interaction can be noticed here with HIS505 (5.68 Å). Unlike lifitegrast, more than four conventional hydrogen bonds were made by hesperidin with residues: ARG403 (3.07 Å), TYR453 (2.95 Å), ARG493 (2.82 Å), SER496 (3.17 Å), TYR501 (3.06 Å), and HIS505 (3.21 Å). In addition, two carbon-hydrogen bonds are formed with SER494 (3.52 Å) and TYR495 (3.41 Å). Concerning other types of interactions, only one pi-alkyl interaction was formed with TYR453 (4.53 Å). Diosmin, with a docking score of − 7.4 kcal/mol, exhibited less variety of interactions as in the case of hesperidin. Diosmin formed four conventional hydrogen bonds SER494 (2.68 Å), SER496 (3.11 Å), and HIS505 (3.17, 3.18 Å). As well, it formed two carbon-hydrogen bonds (ARG403; 3.65 A & TYR495; 3.40 A) and one pi-donor hydrogen bond (TYR501, 3.53 Å). Pi-alkyl interactions (TYR501, 3.78 Å & HIS505, 3.95, 4.74 Å), pi-pi stacked interactions (TYR501, 3.53 Å), and halogen interactions (TYR495, 3.42 Å & HIS505, 3.29 Å) can, also, be observed. Montelukast shows the same number of conventional hydrogen bonds as in lifitegrast and diosmin. Interacting residues that constitute those bonds were as follows: ARG403 (3.67 Å), ASN417 (3.01 Å), TYR453 (3.59 Å), and SER496 (2.24 Å). Moreover, montelukast formed one carbon-hydrogen bond with HIS505 (3.59 Å). For the first time, unfavorable donor-donor and unfavorable acceptor-acceptor interaction occurred with ARG403 (2.13 Å) and GLU406 (2.92 Å), respectively. Further types of interactions can be detected such as pi-pi stacked interactions (TYR501, 4.12 Å & 4.96 Å, HIS505, 4.58 Å), pi-pi T-shaped interactions (HIS505, 5.15 Å), and pi-alkyl interaction (LEU455, 5.08 Å & ARG493, 5.04 Å). Figure S1 specifies the number of hydrogen bonds formed by each residue in O-RBD to show which one contributes the most. In the same way as anti-HIV drugs, SER496 and TYR501 have the main contribution in forming hydrogen bonds.

### Anti-malarial drugs

**Fig. 3:**
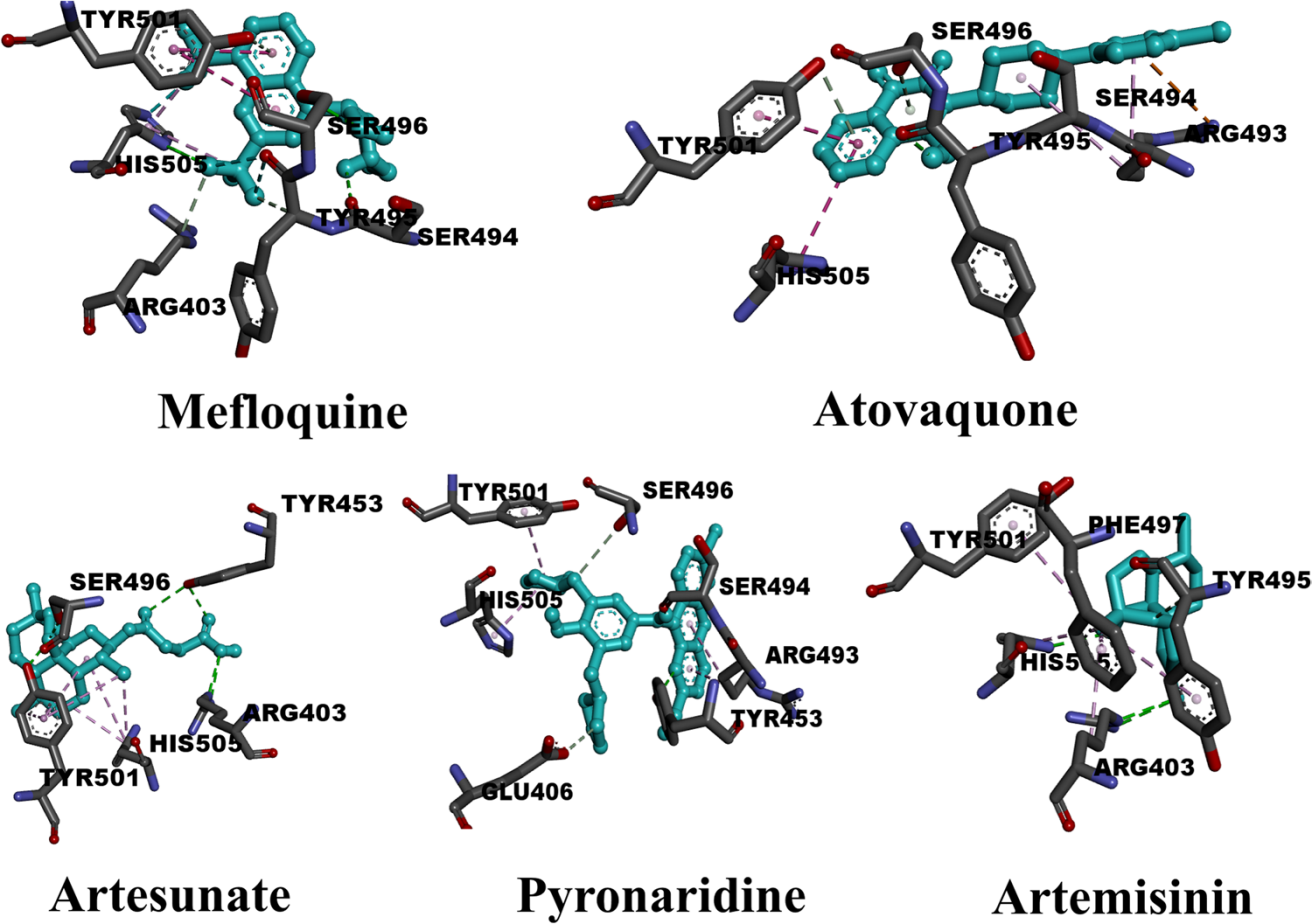
3D representations of binding modes of the best five anti-malarial drugs based on the computed values of docking score

Sixteen anti-malarial agents have been tested as potential inhibitors of the Omicron variant of SARS-CoV-2. The dataset for those compounds is listed in Table S3. The binding features and molecular docking data of the sixteen protein–ligand complexes are summarized in Table S4. From the data in Table S4, all sixteen ligands formed hydrogen bonds of different lengths. The range of scores of molecular docking is from − 5.3 kcal/mol (chloroquine) to − 7.0 kcal/mol (mefloquine). The average value of the docking scores for the sixteen compounds was − 5.95 kcal/mol. Six compounds recorded values above − 6.0 kcal/mol while the other ten compounds recorded values lower than that. For the sake of getting a deep understanding of the other types of interactions in protein–ligand complexes, Fig. 3 shows the full types of interactions for the five highest compounds. What is noticed, mefloquine (− 7.0 kcal/mol) formed three basic types of hydrogen bonds. Four conventional hydrogen bonds with SER494 (2.68), SER496 (3.11 Å), and HIS505 (3.17, 3.18 Å). Two carbon-hydrogen bonds with ARG403 (3.65 Å) and TYR495 (3.40 Å). Pi-donor hydrogen bond with TYR501 (3.53 Å). Residue TYR501 plays a vital role as it formed pi-pi stacked and pi-alkyl interaction with bond lengths equal to 5.31 and 3.78 Å, respectively. Also, HIS505 formed double interaction of pi-alkyl type with lengths equal to 3.95 and 4.47 Å. As mefloquine contains six fluorine atoms, two halogen bonds can be noticed with TYR495 (3.65 Å) and HIS505 (3.29 Å). Atovaquone, the second-highest-ranked compound regarding docking score (− 6.9 kcal/mol), formed three hydrogen bonds. One conventional hydrogen bond with TYR495 (2.30 Å) and two pi-donor hydrogen bonds with SER496 (3.47 Å) and TYR501 (3.70 Å). Remarkably, residue ARG493 formed double interactions with atovaquone that were pi-cation and pi-alky interactions. Also, pi-pi T-shaped interaction can be noticed with HIS505 (5.07 Å). Artesunate scored the highest rate of conventional hydrogen bonds with a docking score of − 6.5 kcal/mol. The interacting residues were ARG403 (4.74 Å), TYR453 (2.96, 3.06 Å), SER496 (2.73 Å), and TYR 501 (2.75 Å). Also, pi-alkyl interaction can be noticed with two residues TYR501 (4.14, 4.92, 5.06, 5.15, 6.05 Å) and HIS505 (4.50, 4.84, 5.28 Å). Although pyronaridine achieved a value of docking score equal to that of artesunate, different types of interactions can be observed. Pyronaridine interacts with the active site of protein through conventional hydrogen bonds with SER494 (1.93 Å) and TYR453 (2.96, 3.21 Å). Similarly, pyronaridine formed two carbon-hydrogen bonds with GLU (3.75 Å) and SER496 (3.69 Å). As observed in the type of interactions that artesunate formed, pyronaridine also favors the pi-alky interaction type. The three residues that make up this type of reaction were ARG493 (4.16, 5.04 Å), TYR501 (4.48 Å), and HIS505 (4.36 Å). Artemisinin, with a value of docking score of − 6.2 kcal/mol, formed three hydrogen bonds. Two of these were conventional hydrogen bonds with ARG403 (3.11 Å) and HIS505 (2.84 Å). The other hydrogen bond is a carbon-hydrogen bond with TYR495 (3.56 Å). Exceptionally, artemisinin formed five bonds of the pi-alky type with a range of bond lengths from 4.80 to 5.37 Å. The degree of interaction in the receptor-ligand complex was determined mostly by hydrogen bonding. As a result, Fig. S1 shows the number of times each class of inhibitor interacted with the O-RBD residues. In the case of inhibitors belonging to an anti-malarial class, SER496 was found to be the main residue of interaction with hydrogen bonds equal to eleven. Residue TYR501 was the second favorable residue with nine hydrogen bonds. Through four hydrogen bonds, anti-malarial drugs interacted with HIS505. Finally, residue ARG498 was identified as the residue with the fewest hydrogen bonds.

### Anti-HIV drugs

**Fig. 4:**
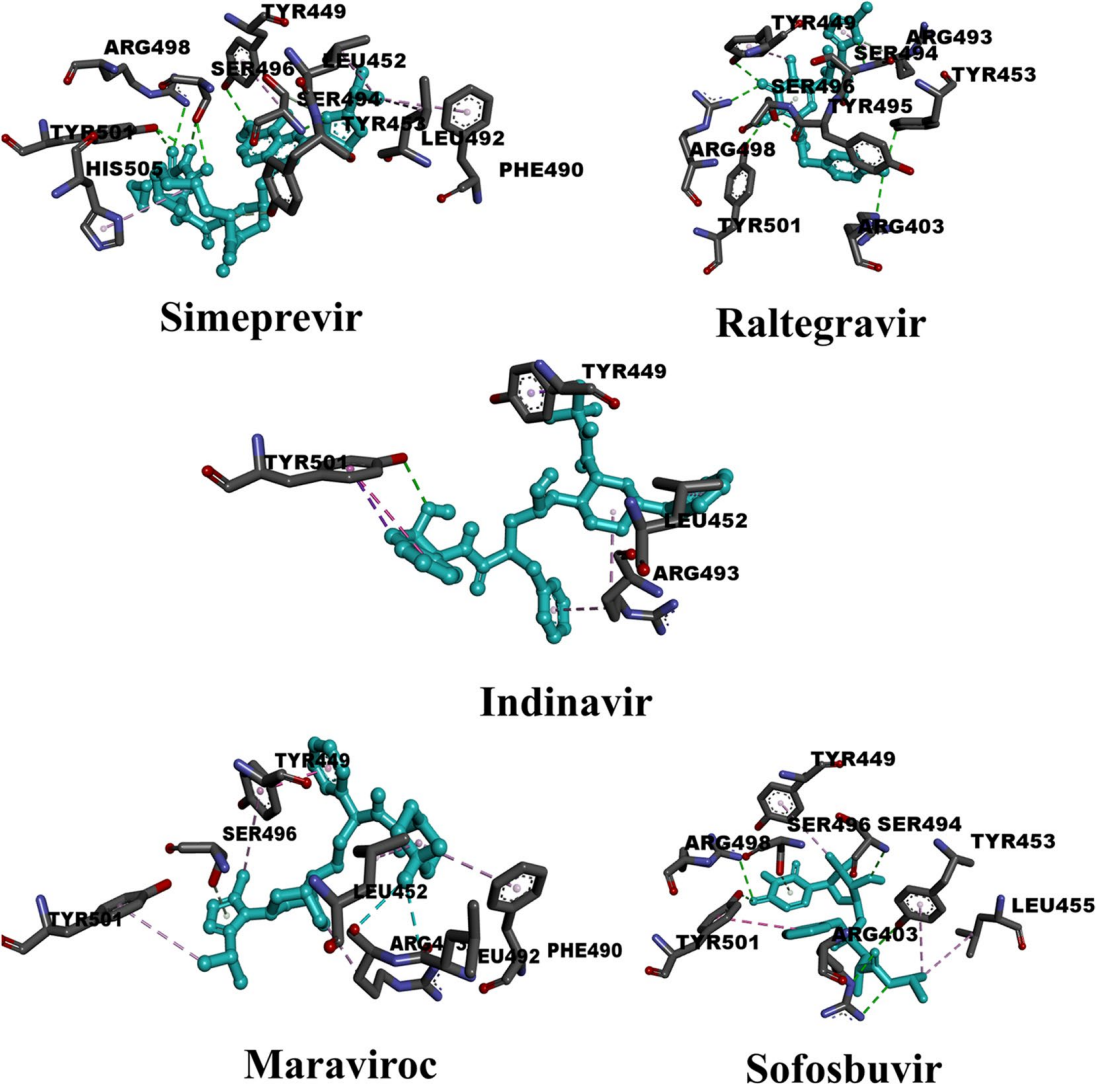
3D representations of binding modes of the best five anti-HIV drugs according to the estimated values of the docking score

Twenty anti-HIV drugs were evaluated for their potential suitability as inhibitors of the recent Omicron variant of SARS-CoV-2. The results (Table S5) showed that the docking scores of twelve of them ranged from − 5.0 to − 6.0 kcal/mol. Ten compounds reached values higher than − 6.0 kcal/mol. Only one compound (Zalcitabine) achieved a value of less than − 5.0 kcal/mol. To gain a deeper insight into the overall validity of the HIV inhibitors, the average docking score for all twenty compounds was calculated and found to be − 5.96 kcal/mol. Table S5 illustrates the binding properties of this class of inhibitors against the Omicron variant of SARS-CoV-2, noting that all inhibitors except indinavir and maraviroc formed more than one hydrogen bond. Figure 4 shows the binding poses of the top five inhibitors within the active site. Interestingly, simeprevir formed multiple conventional hydrogen bonds with SER496 (2.48, 3.05 Å) and TYR501 (2.60, 2.79 Å) and a single conventional hydrogen bond with TYR449 (3.08 Å), SER494 (3.15 Å), and ARG498 (3.23 Å). It also formed a carbon-hydrogen bond with TYR453 (2.77 Å) and many pi-alkyl interactions with LEU452 (4.43, 5.15 Å), PHE490 (4.96 Å), LEU492 (4.82 Å), and HIS505 (5.04 Å). In the case of raltegravir, it formed a large number of conventional hydrogen bonds with TYR449 (2.93 Å), TYR453 (3.11 Å), ARG493 (3.61 Å), SER494 (3.02, 3.25 Å), TYR495 (2.91 Å), SER496 (2.15, 3.98 Å), ARG498 (3.10 Å), and TYR501(2.87 Å). Similar to simeprevir, raltegravir also formed a carbon-hydrogen bond with ARG403 (3.26 Å). In contrast to simeprevir, raltegravir formed only a pi-alky interaction with ARG403 (5.14 Å). As for indinavir, the TYR501 residue formed several types of interactions, such as a conventional hydrogen bond (2.90 Å), pi-pi stacked interaction (5.52 Å), and a pi-sigma interaction (3.79 Å). In addition, indinavir formed one pi-sigma interaction with TYR449 (3.68 Å) and two pi-alkyl interactions with LEU452 (5.06 Å) and ARG493 (4.27, 5.18 Å). Similar to indinavir, maraviroc formed a hydrogen bond, but of the carbon-hydrogen bond type with SER496 (3.07 Å). Since maraviroc contains two fluorine atoms in its structure, a halogen interaction can be observed with LEU492 (3.43 Å) and ARG493 (3.37 Å). The TYR449 residue shows a double interaction with maraviroc through pi-pi stacked interaction (3.92 Å) and a pi-alkyl interaction (5.48 Å). Additional pi-alkyl interactions were detected by maraviroc with the following residues: LEU452 (5.27 Å), PHE490 (5.16 Å), ARG493 (5.48 Å), and TYR501 (5.23 Å). Similar to simeprevir and raltegravir, sofosbuvir shows more than one conventional hydrogen bond and only one carbon-hydrogen bond in its interaction with active site residues. Conventional hydrogen bonds can be detected with the following residues: ARG403 (3.07, 3.20 Å), SER453 (3.03 Å), SER494 (2.90 Å), ARG498 (3.16 Å), and TYR501(2.92 Å). The only carbon-hydrogen bond was with SER496 (3.80 Å). In addition, sofosbuvir formed two other types of interactions: pi-pi stacked interactions with TYR501 (5.44 Å) and pi-alkyl interactions with TYR449 (4.84 Å), TYR453 (4.87 Å), and LEU455 (4.94 Å). Finally, in Fig. S1, we analyzed the most abundant residue in O-RBD with which anti-HIV drugs preferentially interact. Strikingly, HIV inhibitors formed hydrogen bonds fifteen times with SER496, twelve times with TYR501, eleven times with SER494, eight times with ARG498, five times with HIS505, and three times with ARG493. It is clearly apparent that SER496 has the main role rather than other S-RBD residues. The dataset of those compounds is available in Table S6.

### Anti-bacterial drugs

**Fig. 5:**
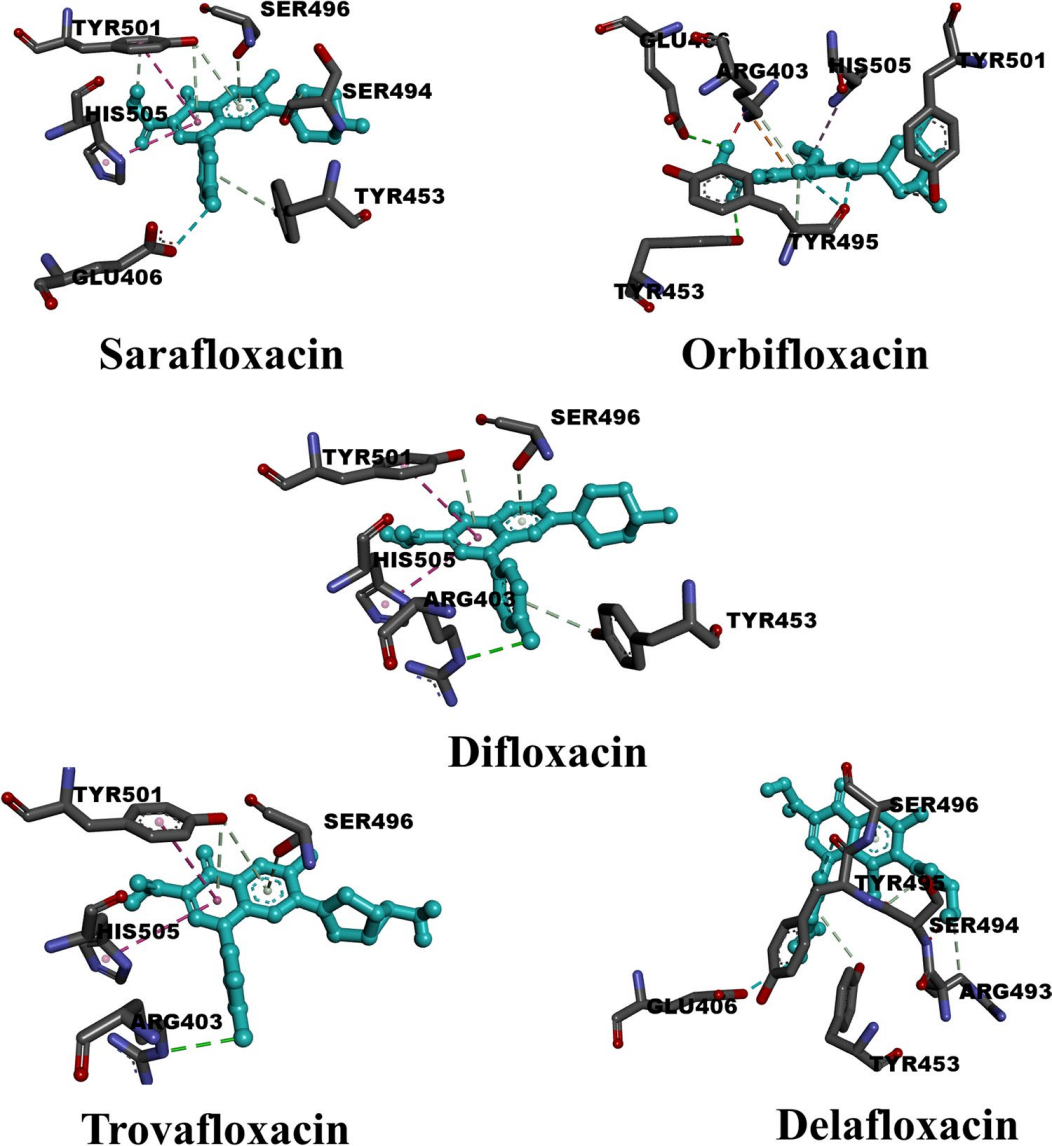
3D representations of binding modes of the best five anti-bacterial drugs according to the estimated values of docking score

In the current study, anti-bacterial agents, particularly fluoroquinolones, were tested to determine their suitability for inhibiting Omicron. The data from the molecular docking study and the binding properties for the twenty compounds tested can be seen in Table S7, respectively. Only two compounds, namely sarafloxacin and difloxacin, achieved docking values greater than − 7.0 kcal/mol. Figure 5 shows the binding pose of the top five drugs. As expected, moderate interactions are observed in contrast to the anti-HIV, anti-inflammatory, and anti-malarial drugs. Sarafloxacin, for example, which has the highest molecular docking score did not record conventional hydrogen bonding with O-RBD residues. Delafloxacin, which ranks fourth in molecular docking score, also does not exhibit conventional hydrogen bonding. In addition, difloxacin and trovafloxacin exhibit only conventional hydrogen bonding with the same residue ARG403. Orbifloxacin is the only fluoroquinolone that forms two conventional hydrogen bonds with GLU406 (2.91 Å) and TYR453 (2.87 Å). Although there were few conventional hydrogen bonds in this class of inhibitors, they instead formed carbon-hydrogen bonds and pi-donor hydrogen bonds. Since all drugs have the fluoroquinolone skeleton, the halogen (fluorine) interactions were the predominant binding features for most of them. Specifically, sarafloxacin formed only one carbon-hydrogen bond with SER494 (3.54 Å). It also formed five pi-donor hydrogen bonds with TYR453 (4.16 Å), SER496 (3.36 Å), and TYR501 (2.69, 4.18, 4.20 Å). Additional types of interactions were observed, such as the pi-pi T-shaped interaction (HIS505, 5.21 Å) and the halogen (fluorine) interaction (GLU406, 3.63 Å). Table S8 comprises the dataset for those compounds.

Similar to sarafloxacin, difloxacin formed a pi-pi T-shaped interaction (HIS505, 5.54 Å) and additionally three pi-donor hydrogen bonds with TYR453 (4.18 Å), SER496 (3.55 Å), and TYR501 (3.99 Å). With a docking score of − 6.80 kcal/mol, trovafloxacin also formed three pi-donor hydrogen bonds with SER496 (3.26 Å) and TYR501 (3.81, 4.06 Å). As with sarafloxacin and difloxacin, residue HIS505 also forms a pi-pi T-shaped interaction with a bond distance of 5.33 Å. No halogen (fluorine) interaction is observed in the interaction of trovafloxacin with O-RBD residues. As in the case of sarafloxacin, delafloxacin also shows pi-donor hydrogen bond interactions (TYR453, 3.95 Å & SER496, 3.48 Å) and carbon-hydrogen bond interactions (ARG493, 3.55 Å & SER494, 3.12 Å). Halogen (fluorine) interactions were registered for residues GLU406 (3.62 Å), SER494 (3.67 Å), and TYR495 (2.80 Å). In contrast to the four aforementioned inhibitors, orbifloxacin exhibited two conventional hydrogen bonds GLU406 (2.91 Å) and TYR453 (2.87 Å). The three remaining hydrogen bonds belonged to the carbon-hydrogen bond type formed with ARG403 (3.50 Å), TYR495 (3.62 Å), and TYR501 (3.52 Å). Other types of interactions can be detected for each residue. For example, a pi-cation interaction (ARG403, 4.12 Å), a pi-alkyl interaction (HIS505, 5.39 Å), and a halogen interaction (TYR495, 2.74 Å). The contribution of each residue in the O-RBD is shown in Fig. S1. Only three residues were found to form hydrogen bonds with fluoroquinolones. As with the previous classes, SER496 and TYR501 had the most contributions, each with an equal number of hydrogen bonds. Only one hydrogen bond was formed by ARG493.

### In silico evaluation of molecular properties

OSIRIS Property Explorer was used to calculate drug-related characteristics such as toxicity risks, drug-likeness score, cLogP, logS, and MW. All of these parameters provide an indication of an important property of the drug under study. For example, the cLogP parameter gives an indication of lipophilicity. Similarly, the logS parameter gives an indication of hydrophilicity. As highlighted in the methodology section, Osiris has an important feature in that it can combine all these parameters into a single value called the drug score. This type of analysis was performed for the five compounds with the highest docking scores from each of the four classes. MD simulations were only performed for compounds that had a positive drug-likeness score and the highest drug score compared to their analogs from the same class.

### Anti-HIV drugs

**Fig. 6:**
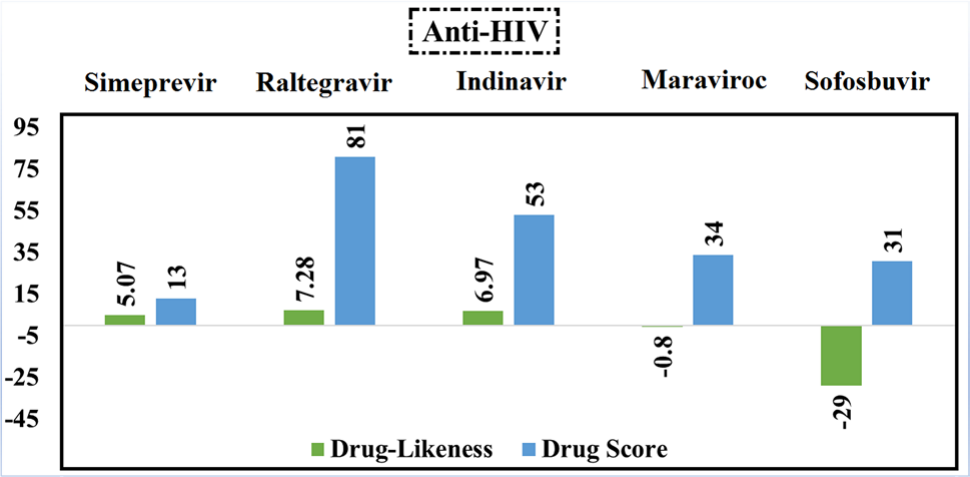
Calculated values of drug-likeness and drug score of the best five anti-HIV compounds based on the data of molecular docking study

All anti-HIV drugs showed positive values of the drug-likeness score except maraviroc and sofosbuvir (Fig. 6). Consequently, maraviroc and sofosbuvir were excluded. Regarding values of drug score, raltegravir obtained the highest value, which is equal to 81%. So, raltegravir was considered for further MD simulations.

### Anti-inflammatory drugs

**Fig. 7:**
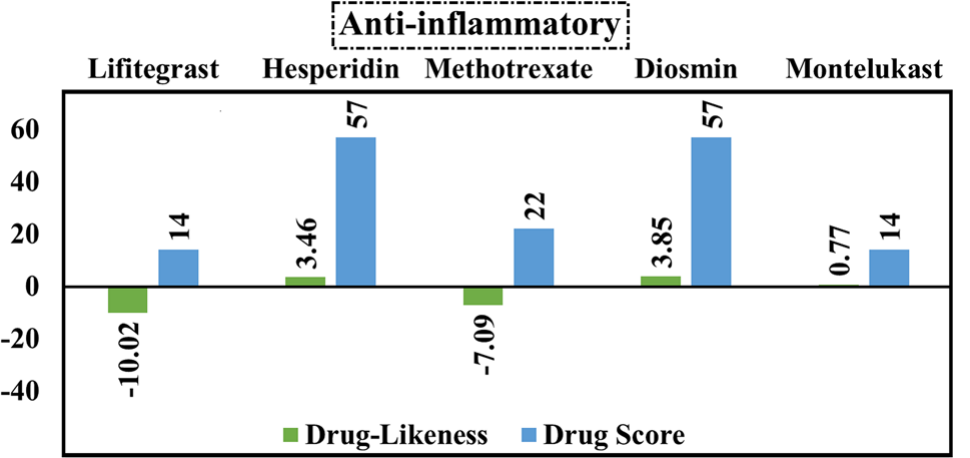
Calculated values of drug-likeness and drug score of the best five anti-inflammatory compounds based on the data of molecular docking study

Excluding lifitegrast and methotrexate, all anti-inflammatory drugs exhibited positive drug-likeness scores (Fig. 7). As can be observed from the data of anti-inflammatory drugs in Fig. 7, lifitegrast and montelukast achieved the same value of drug score (14%). In addition, the same drug score value (57%) was estimated for hesperidin and diosmin. According to the calculated data in the molecular docking study, hesperidin has an advantage over diosmin with a docking score of − 7.8 kcal/mol. As a consequence, hesperidin was selected for the MD simulation study.

### Anti-malarial drugs

**Fig. 8:**
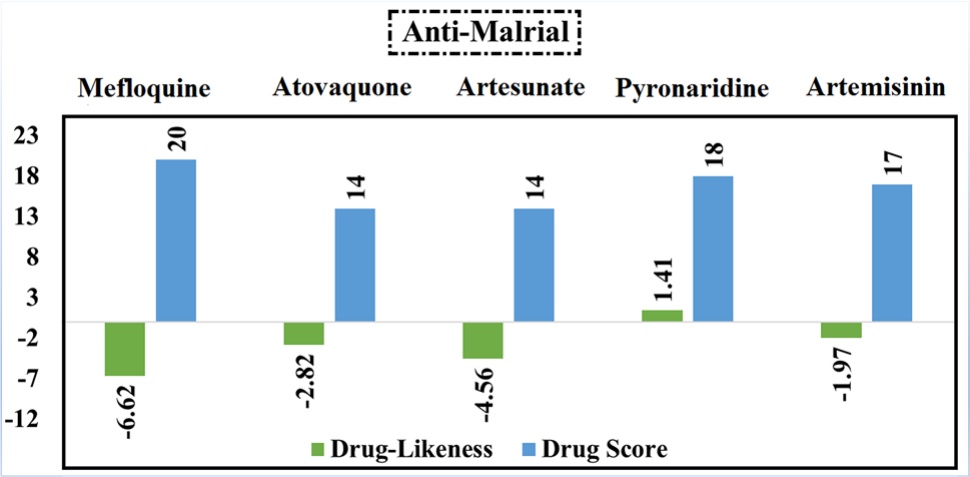
Calculated values of drug-likeness and drug score of the best five anti-malarial compounds based on the data of molecular docking study

What is important about the data on anti-malarial drugs (Fig. 8) is that all inhibitors achieved negative drug-likeness scores except pyronaridine. Although mefloquine has a value of drug score equal to 20%, pyronaridine (DS = 18%) was chosen for the MD simulation study as it has a positive value of drug-likeness which is equal to 1.41.

### Anti-bacterial agents

**Fig. 9:**
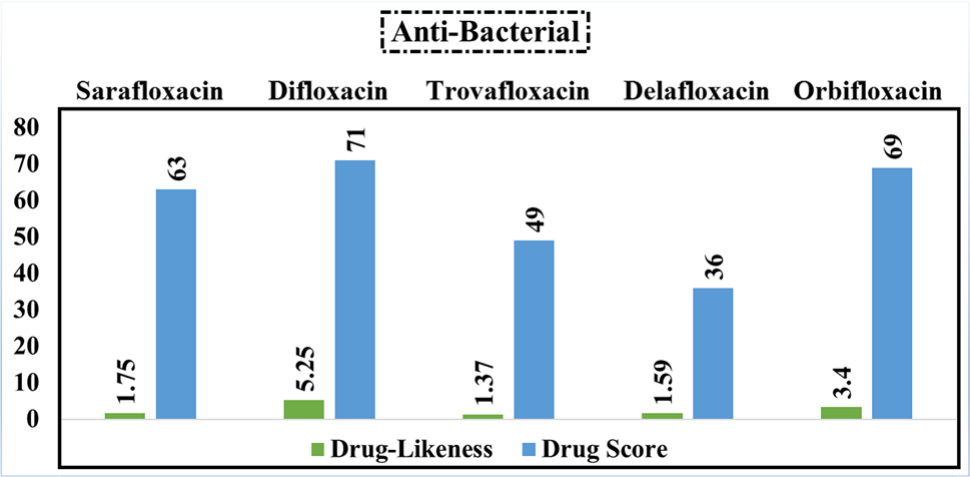
Calculated values of drug-likeness and drug score of the best five anti-bacterial compounds based on the data of molecular docking study

It is worth mentioning that all inhibitors belonging to the anti-bacterial class exhibit positive values for drug-likeness (Fig. 9). Difloxacin obtained the highest value of both drug-likeness (5.25) and drug score (71%). Accordingly, it was considered in performing the MD simulation study.

### Molecular dynamics (MD) simulations

The purpose of using MD simulations is attributed to its main role in studying conformational stability and obtaining reliable results about the behavior of the studied compounds in the active site of the protein. In accordance with the results of the molecular docking study and the review of molecular properties, the best compound from each class was selected. MM/GBSA binding energy calculations studied four complexes over 100 ns MD simulations to estimate the binding behavior of the ligand to the receptor. The calculated MM/GBSA binding energies of the four protein–ligand complexes over 100 ns are shown in Table 1.

**Table 1:**
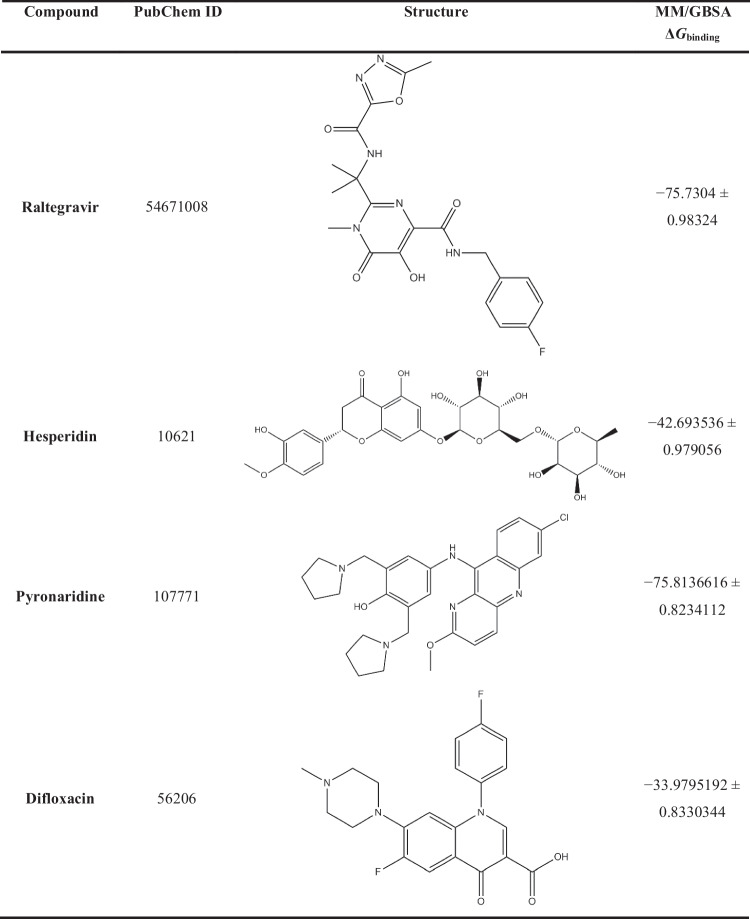
Calculated MM/GBSA binding energies (in kJ/mol) over 100 ns for the best compound in each investigated class.

What is noticed from the data shown in Table 1 is that difloxacin, which has a docking score and drug score of − 7.0 kcal/mol and 71.0%, respectively, obtained the lowest value of Δ*G*_binding_ of − 33.9795192 ± 0.8330344 kJ/mol which reflects its weak inhibition of SARS-CoV-2 Omicron. Taking into consideration the best drug from the anti-HIV class, the computed MM/GBSA binding energies revealed that raltegravir scored adequate inhibition of SARS-CoV-2 Omicron with Δ*G*_binding_ of − 75.7304 ± 0.98324 kJ/mol. Strikingly, pyronaridine, an anti-malarial drug, scored Δ*G*_binding_ of − 75.8136616 ± 0.8234112, which is very close to the value of raltegravir. The best anti-inflammatory drug, hesperidin, showed relatively high binding affinity towards SARS-CoV-2 Omicron with a Δ*G*_binding_ of − 42.693536 ± 0.979056 kJ/mol.

For quantitative assessment of the overall stability of raltegravir, hesperidin, pyronaridine, and difloxacin complexed with SARS-CoV-2 Omicron over 100 ns MD simulations, analyses of root-mean-square deviation (RMSD), root-mean-square fluctuation (RMSF), radius of gyration (Rg), and solvent-accessible surface area (SASA) were performed.

**Fig. 10:**
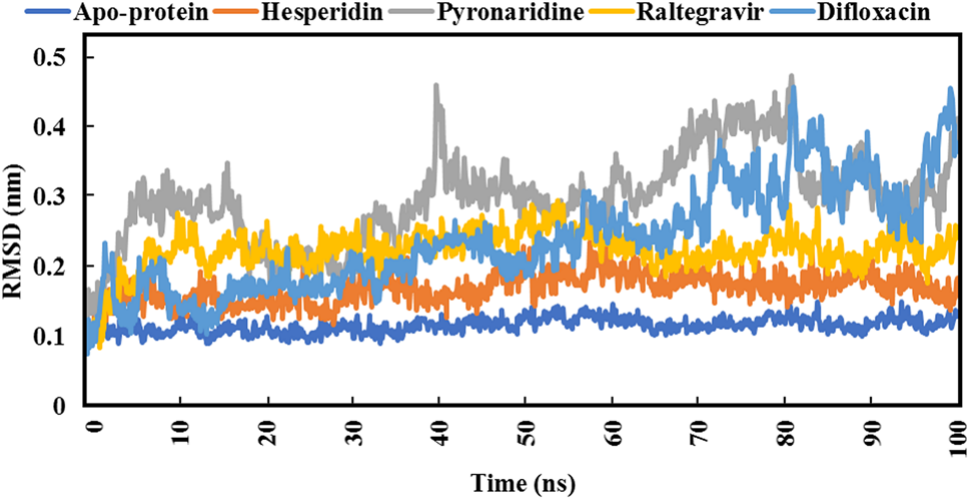
Root-mean-square deviation (RMSD) SARS-CoV-2 Omicron backbone atoms from the initial structure complexed with the highest-ranked compounds over 100 ns MD simulations

The RMSD for the four complexes under investigation was calculated to assess conformational change and the systems’ stability during the 100 ns MD simulation periods [77]. Higher values of RMSD demonstrate relative instability, whereas lower RMSD values are more favorable. The RMSD plot for the four simulation systems can be witnessed in Fig. 10.

As depicted in Fig. 10, pyronaridine exhibited higher RMSD values as compared to others, which indicates its moderate stability inside the SARS-CoV-2 Omicron active site. Besides, hesperidin, with a Δ*G*_binding_ equal to − 42.693536 ± 0.979056 kJ/mol, showed values of RMSD less than 0.21 nm. With respect to raltegravir, values of RMSD over the 100 ns MD simulations were less than 0.28 nm, which is compatible with the estimated value of binding energy (− 75.7304 ± 0.98324 kJ/mol). Difloxacin, the lowest compound out of the four in terms of binding energy (− 33.9795192 ± 0.8330344 kJ/mol), exhibited relative instability from 70 to 100 ns. The average RMSD value of the apoprotein structure was 0.13 nm.

**Fig. 11:**
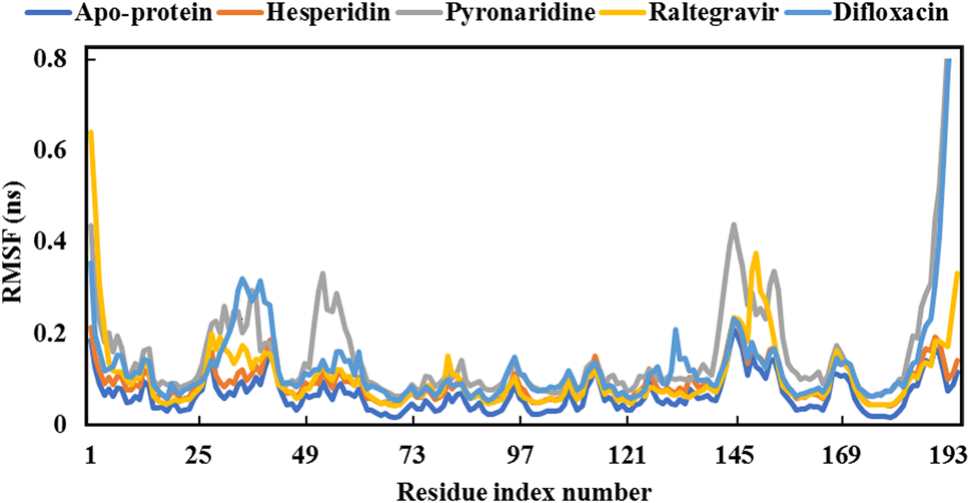
Root-mean-square fluctuation (RMSF) of the apoprotein structure and five selected complexes

RMSF was employed with the aim of identifying the flexibility and fluctuation of each SARS-CoV-2 Omicron residue, in addition to how much the movement of each residue over the whole simulation period. The flexibility of each residue was investigated in an attempt to better comprehend how ligand binding influences protein flexibility during the MD simulation course. Lower values of RMSF imply better receptor compactness, stiffness, and stability. The RMSF values for the four studied complexes are figured out in Fig. 11.

What is noticed from the data in Fig. 11 is results of RMSF were nearly compatible with RMSD data. Difloxacin and pyronaridine displayed relatively high values of RMSF, which indicate the high amino acid residues’ fluctuation when complexed with those inhibitors. It is worth mentioning that raltegravir, as well as hesperidin, showed similar values of RMSF, i.e., a similar effect on fluctuations of amino acid residues. The average RMSF value of the apoprotein structure was 0.065 nm.

**Fig. 12:**
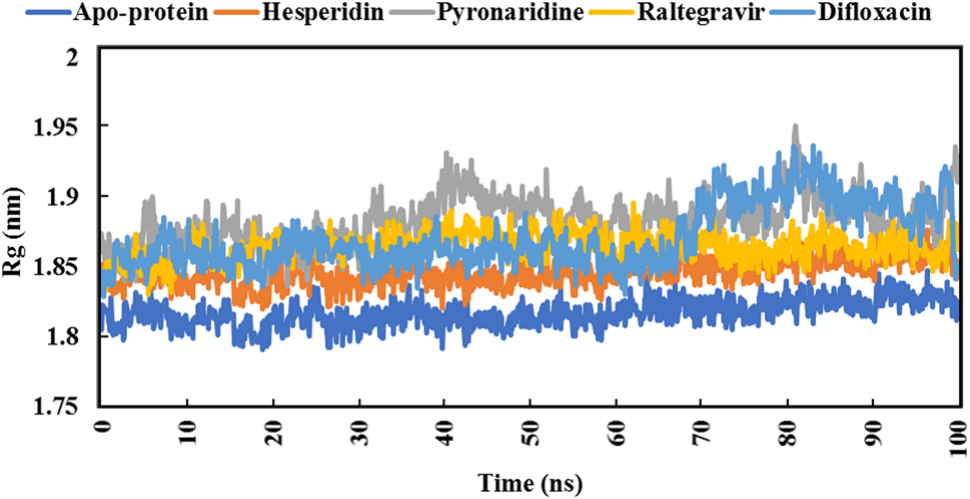
Radius of gyration (Rg) plot of apoprotein structure and five identified complexes through 100 ns MD simulations

The Rg analysis was accomplished over 100 ns MD simulations to interpret the compactness of the protein structure inside the system [78]. Low Rg values indicate conformational stability and the degree to which the protein structure is tightly packed. Rg data is illustrated in Fig. 12.

From the beginning of 70 ns to the end of the MD simulation, raltegravir showed very close values to hesperidin. Moreover, also from 70 to 100 ns, pyronaridine and difloxacin showed remarkable overlap in Rg values. The average Rg value of the apoprotein structure was 1.81 nm.

**Fig. 13:**
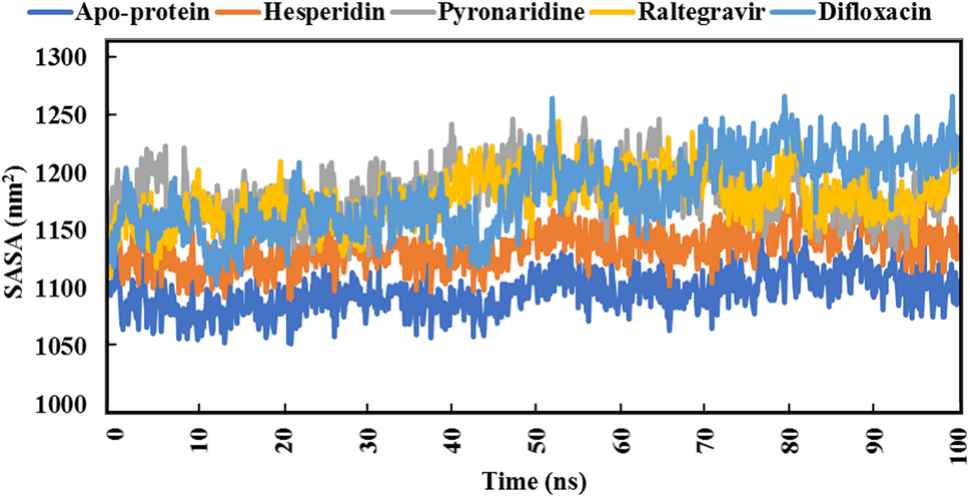
Solvent-accessible surface area (SASA) of apoprotein structure and the best five compounds for 100 ns MD simulations

SASA is employed to specify the surface area of the receptor that is available to a solvent [79, 80]. Figure 13 represents the data of SASA of the four compounds.

Estimated results demonstrated that hesperidin exhibited the lowest values of SASA as compared to others. On the other side, difloxacin showed an extensive increase in SASA values from 70 ns to the end of the MD simulation period. Both raltegravir and pyronaridine showed overlapping SASA values over the whole MD simulation period. The average SASA value of the apoprotein structure was 1097.15 nm^2^.

## Limitations of this study

The limitations of this study are as follows:

1. The 100 ns scale molecular dynamics simulation may be short to evaluate the stability of identified compounds inside the cavity, but unfortunately, we do not have enough devices to extend the simulation time up to 1,000,000 ns.
2. As the study was established based on drug repurposing methodology, there are previous experimental assays available on the FDA database for most of the investigated drugs. It is worth mentioning that the present study relied on estimating the physicochemical and toxicological molecular properties.
3. Other classes of inhibitors should be investigated as anti-Omicron drugs. It is worth mentioning that the current study addresses the potential activity of four classes of inhibitors, anti-HIV, anti-malarial, anti-inflammatory, and anti-bacterial, which were mentioned in the previous studies as anti-COVID-19 drugs.

## Summary and conclusions

The emergence of SARS-CoV-2 Omicron led to increased viral spread and transmissibility of the virus. Amino acid variations, particularly in the RBD, are a prominent feature of Omicron. A total of seventy-one compounds from four different classes were evaluated in detail. In this study, molecular docking, prediction of drug-relevant properties using Osiris property explorer, and MD simulations were used to select the best compound in each class. According to the results, raltegravir is the best HIV inhibitor with a drug score and Δ*G*_binding_ of 81% and − 75.7304 ± 0.98324 kJ/mol, respectively. Hesperidin (anti-inflammatory) also showed a high inhibition rate towards Omicron with a drug score and Δ*G*_binding_ of 57% and − 42.693536 ± 0.979056 kJ/mol, respectively. On the other hand, moderate inhibition of pyronaridine (an anti-malarial) and difloxacin (anti-bacterial) was observed. The current results suggest that raltegravir and hesperidin may have potential activity against the SARS-CoV-2 Omicron variant and should be considered for further in vivo and in vitro studies.

## Supplementary information

Below is the link to the electronic supplementary material.

Supplementary file1 (DOCX 236 KB)

## Data Availability

The datasets supporting this article have been uploaded as part of the electronic supplementary material.
