# Supplementary material for: In silico prediction of potential inhibitors for SARS-CoV-2 Omicron variant using molecular docking and dynamics simulation-based drug repurposing
Source: J Mol Model. 2023 Feb 20;29(3):70. doi: 10.1007/s00894-023-05457-z (PMC9939377; doi:10.1007/s00894-023-05457-z)
Supplement: Supplementary file 1 — Supplementary file1 (DOCX 236 KB) [file 894_2023_5457_MOESM1_ESM.docx]

*In silico* prediction of potential inhibitors for SARS-CoV-2 omicron variant using molecular docking and dynamics simulation-based drug-repurposing

Eslam A.R. Mohamed^1^, Islam M. Abdel-Rahman^2^, Magdi E. A. Zaki^3, *^, Ahmad Al-Khdhairawi^4^, Mahmoud M. Abdelhamid^5^, Ahmad M. Alqaisi^6^, Lyana binti Abd Rahim^7^, Bilal Abu-Hussein^8^, Azza A.K. El-Sheikh^9^, Sayed F. Abdelwahab^10^, Heba Ali Hassan^11, *^

^1^ Department of Chemistry, Faculty of Science, Minia University, Minia, 61511, Egypt; [eslamahmedragabmohamed@gmail.com](mailto:eslamahmedragabmohamed@gmail.com)

^2^ Department of Pharmaceutical Chemistry, Faculty of Pharmacy, Deraya University, New-Minia, 61519-Minia, Egypt; [dr.islammoh@deraya.edu.eg](mailto:dr.islammoh@deraya.edu.eg)

^3^ Department of Chemistry, Faculty of Science, Imam Mohammad Ibn Saud Islamic University (IMSIU), Riyadh, Saudi Arabia; [mezaki@imamu.edu.sa](mailto:mezaki@imamu.edu.sa)

^4^ Department of Biological Science and Biotechnology, Faculty of Science and Technology, Universiti Ke-bangsaan Malaysia, 43600 UKM Bangi, Selangor, Malaysia; [ahmadayad@outlook.my](mailto:ahmadayad@outlook.my)

^5^ Department of pharmaceutical Chemistry, Faculty of Pharmacy, Al-Azhar University, Assiut 71524, Egypt; [mahmod.albakry@gmail.com](mailto:mahmod.albakry@gmail.com)

^6^ Chemistry Department, University of Jordan, Amman, 11942, Jordan; [alqigjika2008@gmail.com](mailto:alqigjika2008@gmail.com)

^7^ Department of Medicine, Hospital Tuanku Ampuan Najihah, Kuala Pilah, Negeri Sembilan, Malaysia; [lyanaabd.rahim@yahoo.com](mailto:lyanaabd.rahim@yahoo.com)

^8^ Albayader Specialty Hospital, Amman, Jordan; [asad.bilaldr@gmail.com](mailto:asad.bilaldr@gmail.com)

^9^ Basic Health Sciences Department, College of Medicine, Princess Nourah bint Abdulrahman University, P.O. 13 Box 84428, Riyadh 11671, Saudi Arabia. [aaelsheikh@pnu.edu.sa](mailto:aaelsheikh@pnu.edu.sa)

^10^ Department of Pharmaceutics and Industrial Pharmacy, College of Pharmacy, Taif University, PO Box 11099, Taif 21944, Saudi Arabia. [s.fekry@tu.edu.sa](mailto:s.fekry@tu.edu.sa)

^11^ Department of Pharmacognosy, Faculty of Pharmacy, Sohag University, Sohag 82524, Egypt; [heba.ali@pharm.sohag.edu.eg](mailto:heba.ali@pharm.sohag.edu.eg)

* **Correspondence to**:

[heba.ali@pharm.sohag.edu.eg](mailto:heba.ali@pharm.sohag.edu.eg)

**Table S1** Compound names, docking scores (in kcal/mol), and binding features of fifteen anti-inflammatory compounds against Omicron variant of SARS-Cov-2.

| No | Compound | Docking Score (kcal/mol) | Binding Features |
| --- | --- | --- | --- |
| 1 | Lifitegrast | −8.1 | ARG403 (2.89 Å), TYR453 (4.05 Å),  , SER496 (2.90, 2.98 Å), TYR501 (2.90 Å) |
| 2 | Hesperidin | −7.8 | ARG403 (3.07 Å), ARG493 (2.82 Å), SER494 (3.52 Å), TYR495 (3.41 Å), TYR453 (2.95 Å), SER496 (3.17 Å), TYR501 (3.06 Å), HIS505 (3.21 Å) |
| 3 | Diosmin | −7.4 | ARG403 (3.65 Å), SER494 (2.68 Å), TYR495 (3.40 Å), SER496 (3.11 Å), TYR501 (3.53 Å), HIS505 (3.17, 3.18 Å) |
| 4 | Montelukast | −7.1 | ARG403 (3.67 Å) ASN417 (3.01 Å), TYR453 (3.59 Å), SER496 (2.24 Å), HIS505 (3.59 Å) |
| 5 | Methotrexate | −6.6 | TYR453 (2.63 Å), LEU492 (2.02 Å), SER494 (2.00, 2.63 Å), SER496 (3.26, 4.07 Å), TYR501 (3.28 Å) |
| 6 | Antrafenine | −6.6 | ARG403 (3.25 Å), TYR453 (3.09 Å), HIS505 (3.14, 3.62 Å) |
| 7 | Prednisolone | −6.5 | TYR495 (2.76 Å), SER496 (2.74 Å), TYR501 (3.18 Å), HIS505 (2.63 Å) |
| 8 | Piroxicam | −6.5 | SER496 (3.06 Å), ARG498 (3.02 Å), TYR501 (2.97, 3.63 Å) |
| 9 | Baricitinib | −6.1 | ARG403 (2.99, 3.15 Å), SER496 (2.95 Å), TYR501 (3.86 Å) |
| 10 | Ruxolitinib | −6.1 | ARG403 (3.68 Å), ARG493 (3.44 Å), SER496 (3.23 Å) |
| 11 | tofacitinib | −6 | ARG403 (3.65 Å), TYR453(3.32 Å), ARG493 (3.46 Å), SER494 (2.88, 3.50 Å) |
| 12 | lenalidomide | −5.9 | ARG493 (3.38 Å), SER494 (3.04 Å), SER496 (2.88 Å), TYR501 (3.88 Å) |
| 13 | Dexamethasone | −5.9 | ASN417 (2.91 Å), HIS505 (3.47 Å) |
| 14 | Thalidomide | −5.8 | ARG493 (3.49 Å), SER494 (2.92 Å), SER496 (2.84 Å), TYR501 (3.83 Å) |
| 15 | colchicine | −5.3 | ARG403 (3.01 Å), GLU406 (3.42 Å), TYR453 (3.23, 3.66 Å) |

^a^ Conventional hydrogen bonds, carbon-hydrogen bonds, and pi-donor hydrogen bonds (in Å) were listed.

**Table S2** Dataset of the studied anti-inflammatory agents, including PubChem ID, pharmacological indication, and group.

| **No** | **Compound** | **PubChem ID** | **Indication** | **Group** |
| --- | --- | --- | --- | --- |
| 1 | Lifitegrast | 11965427 | It inhibits T-cell adhesion to ICAM-1 in a human T-cell line and may inhibit secretion of inflammatory cytokines, inflammatory mediators, chemokines, TNF-α, and IL-1 in human peripheral blood mononuclear cells. | Approved |
| 2 | Hesperidin | 10621 | A natural flavonoid that possesses anti-inflammatory properties in many disease models. | Approved, Investigational |
| 3 | Diosmin | 3002977 | Anti-inflammatory and antiradical effects | Approved, Investigational |
| 4 | Montelukast | 5281040 | A systemically active drug with a targeted, dual mechanism of action, acting both as a bronchodilator and anti-inflammatory | Approved |
| 5 | Methotrexate | 126941 | Inhibits enzymes responsible for nucleotide synthesis which prevents cell division and leads to anti-inflammatory actions | Approved |
| 6 | Antrafenine | 68723 | An analgesic and anti-inflammatory drug with similar efficacy to naproxen | Approved |
| 7 | Prednisolone | 5755 | A glucocorticoid used to treat adrenocortical insufficiency, inflammatory conditions, and some cancers | Approved |
| 8 | Piroxicam | 54676228 | non-steroidal anti-inflammatory agent (NSAID) that is well established in treating rheumatoid arthritis and osteoarthritis and used for musculoskeletal disorders, dysmenorrhea, and postoperative pain | Approved, Investigational |
| 9 | Baricitinib | 44205240 | tyrosine protein kinases that play an important role in pro-inflammatory signaling pathways | Approved, Investigational |
| 10 | Ruxolitinib | 25126798 | The topical formulation of ruxolitinib is used to treat atopic dermatitis and vitiligo.23 It is being investigated for other inflammatory skin conditions | Approved |
| 11 | Tofacitinib | 9926791 | Tofacitinib targets inflammation present in rheumatoid arthritis by inhibiting the janus kinases involved in the inflammatory response pathway | Approved, Investigational |
| 12 | Lenalidomide | 216326 | an immunomodulatory drug with potent antineoplastic, anti-angiogenic, and anti-inflammatory properties | Approved |
| 13 | Dexamethasone | 5743 | a glucocorticoid available in various modes of administration that is used for the treatment of various inflammatory conditions, including bronchial asthma, as well as endocrine and rheumatic disorders | Approved, Investigational |
| 14 | Thalidomide | 5426 | used for a number of inflammatory disorders and cancers | Approved, Investigational |
| 15 | colchicine | 6167 | an alkaloid used in the symptomatic relief of pain in attacks of gout and to treat the inflammatory symptoms of Familial Mediterranean Fever (FMF) | Approved |

**Table S3** Dataset of the studied anti-malarial agents, including PubChem ID, pharmacological indication, and group.

| **No** | **Compound** | **PubChem ID** | **Indication** |  |
| --- | --- | --- | --- | --- |
| 1 | Mefloquine | 4046 | An antimalarial agent used in the prophylaxis and treatment of malaria caused by Plasmodium falciparum and Plasmodium vivax | Approved, Investigational |
| 2 | Atovaquone | 74989 | For the prevention and treatment of Plasmodium falciparum malaria | Approved |
| 3 | Artesunate | 105031 | An artemesinin derivative indicated for the initial treatment of severe malaria | Approved, Investigational |
| 4 | Pyronaridine | 107771 | A benzonaphthyridine derivative indicated in the treatment of acute malaria caused by P. falciparum or P. vivax in area with low rates of infection and low resistance to artemisinin | Investigational |
| 5 | Artemisinin | 68827 | A medication indicated in the treatment of malaria | Investigational |
| 6 | Pyrimethamine | 4993 | An antiparasitic drug used in the prevention and treatment of toxoplasmosis and malaria | Approved, Investigational |
| 7 | Artenimol | 107770 | An artemisinin derivative and antimalarial agent used in the treatment of uncomplicated Plasmodium falciparum infections | Approved, Experimental, Investigational |
| 8 | Artemether | 68911 | An antimalarial agent used in combination with lumefantrine for the treatment of acute uncomplicated malaria caused by Plasmodium falciparum | Approved |
| 9 | Amodiaquine | 2165 | An aminoquinoline used for the therapy of malaria | Approved, Investigational |
| 10 | Quinine | 3034034 | An alkaloid used to treat uncomplicated Plasmodium falciparum malaria | Approved |
| 11 | Sulfadoxine | 17134 | A long acting sulfonamide used for the treatment or prevention of malaria | Approved, Investigational |
| 12 | Lumefantrine | 6437380 | An antimalarial agent used in combination with artemether for the treatment of acute uncomplicated malaria caused by Plasmodium falciparum | Approved |
| 13 | Halofantrine | 37393 | An antimalarial used for the treatment of severe malaria | Approved |
| 14 | Proguanil | 6178111 | A medication indicated for prophylaxis and treatment of Plasmodium falciparum malaria | Approved |
| 15 | Hydroxychloroquine | 3652 | An antimalarial medication used to treat uncomplicated cases of malaria and for chemoprophylaxis in specific regions | Approved |
| 16 | Chloroquine | 2719 | An antimalarial drug used to treat susceptible infections with P. vivax, P. malariae, P. ovale, and P. falciparum. | Approved, Investigational, |

**Table S4** Compound names, docking scores (kcal/mol), and binding features of anti-malarial agents against SARS-Cov-2 Omicron variant.

| **No** | **Compound** | **Docking Score (kcal/mol)** | **Binding Features^a^** |
| --- | --- | --- | --- |
| 1 | Mefloquine | −7.0 | ARG403 (3.65 Å), SER494 (2.68 Å), TYR495 (3.40 Å), SER496 (3.11 Å), TYR501 (3.53 Å), HIS505 (3.17, 3.18 Å) |
| 2 | Atovaquone | −6.9 | TYR495 (2.30 Å), SER496 (3.47 Å), TYR501 (3.70 Å) |
| 3 | Artesunate | −6.5 | ARG403 (4.74 Å), TYR453 (2.96, 3.06 Å), SER496 (2.73 Å), TYR 501 (2.75 Å) |
| 4 | Pyronaridine | −6.5 | GLU406 (3.75 Å), TYR453 (2.96, 3.21 Å), SER494 (1.93 Å), SER496 (3.69 Å) |
| 5 | Artemisinin | −6.2 | ARG403 (3.11, 3.27 Å), TYR495 (3.56 Å)), HIS505 (2.84 Å) |
| 6 | Pyrimethamine | −6.1 | TYR453 (2.48 Å) |
| 7 | Artenimol | −5.9 | HIS505(2.90 Å), ARG403 (3.05, 3.18 Å), GLU406 (2.39 Å) |
| 8 | Artemether | −5.8 | HIS505(3.71 Å) |
| 9 | Amodiaquine | −5.7 | TYR453(3.01 Å) |
| 10 | Quinine | −5.7 | SER496 (3.54 Å), TYR505 (3.56, 3.72 Å) |
| 11 | Sulfadoxine | −5.7 | ARG403 (3.19 Å), TYR453 (3.23 Å), GLU406 (3.79 Å), SER496 (2.93 Å), TYR505 (3.23 Å) |
| 12 | Lumefantrine | −5.6 | TYR495 (2.21 Å), SER496 (2.74 Å), TYR (3.31, 3.72, 4.05 Å) |
| 13 | Halofantrine | −5.5 | SER496 (3.33 Å), TYR501 (3.64, 3.80 Å) |
| 14 | Proguanil | −5.4 | SER494 (2.17 Å), TYR495 (2.02, 2.50 Å), SER496 (2.75, 2.78 Å), TYR501 (3.66 Å) |
| 15 | Hydroxychloroquine | −5.4 | TYR453 (2.79 Å), SER494 (1.95 A), TYR495 (2.09 Å), SER496 (3.09 Å) TYR501 (3.88, 4.16 Å) |
| 16 | Chloroquine | −5.3 | TYR501 (3.39 Å) |

^a^ Conventional hydrogen bonds, carbon-hydrogen bonds, and pi-donor hydrogen bonds (in Å) were listed.

**Table S5** Compound names, docking scores (kcal/mol), and binding features of twenty HIV inhibitors against Omicron variant of SARS-Cov-2.

| No | Compound | Docking Score (kcal/mol) | Binding Features^a^ |
| --- | --- | --- | --- |
| 1 | Simeprevir | −8.3 | TYR449 (3.08 Å), TYR453 (2.77 Å), SER494 (3.15 Å), SER496 (2.48, 3.05 Å), ARG498 (3.23 Å), TYR501 (2.60, 2.79 Å) |
| 2 | Raltegravir | −7.4 | ARG403 (3.26 Å), TYR449 (2.93 Å), TYR453 (3.11 Å), ARG493 (3.61 Å), SER494 (3.02, 3.25 Å), TYR495 (2.91 Å), SER496 (2.15, 3.98 Å), ARG498 (3.10 Å), TYR501 (2.87 Å) |
| 3 | Indinavir | −7.2 | TYR501 (2.90 Å) |
| 4 | Maraviroc | −7.1 | SER496 (3.07 Å) |
| 5 | Sofosbuvir | −6.9 | ARG403 (3.07, 3.20 Å), TYR453 (3.03 Å), SER494 (2.90 Å), SER496 (3.80 Å), ARG498 (3.16 Å), TYR501 (2.92 Å) |
| 6 | Nelfinavir | −6.5 | TYR449 (3.99 Å), SER494 (2.11, 2.33, 2.86, 3.09 Å) |
| 7 | Amprenavir | −6.3 | TYR449 (2.81 Å), TYR453 (2.86, 3.30 Å), TYR495 (2.94, 3.07 Å), SER496 (2.58 Å), ARG498 (3.04, 3.10 Å), HIS505 (2.52 Å) |
| 8 | Delavirdine | −6.3 | ARG403 (3.02, 3.14 Å), TYR453 (2.87 Å), SER494 (3.10 Å), TYR495 (2.26 Å), SER496 (2.10, 3.08, 3.10 Å) |
| 9 | Darunavir | −6.1 | TYR453 (2.74, 3.55 Å), TYR495 (2.58 Å), SER496 (3.04 Å), ARG498 (3.32 Å), TYR501 (2.97 Å), HIS505 (2.55 Å) |
| 10 | Efavirenz | −6 | TYR495 (3.20 Å), SER496 (2.95, 3.09 Å), HIS505 (3.19 Å) |
| 11 | Elvitegravir | −5.8 | TYR449 (2.38 Å), TYR453 (3.64 Å)  SER494 (2.92, 3.64 Å), ARG498 (3.25 Å), SER496 (3.68, 3.78 Å), TYR501 (3.14 Å), |
| 12 | Nevirapine | −5.8 | TYR453 (3.59 Å), SER494 (3.55 Å) |
| 13 | Ritonavir | −5.7 | GLU406 (3.39 Å)  ARG403 (3.13, 3.31 Å), ASN417 (3.73 Å), TYR453 (2.36, 2.78, 3.61 Å), SER494 (2.23, 3.36 Å), ARG498 (3.20 Å), TYR501 (2.97 Å) |
| 14 | Sequinavir | −5.5 | SER496 (3.03 Å), TYR501 (3.19 Å) |
| 15 | Stavudine | −5.3 | TYR453 (3.34 Å), SER494 (3.55 Å), TYR501 (3.79 Å) |
| 16 | Lopinavir | −5.2 | TYR453 (2.35, 2.74, 3.04 Å), ARG493 (3.72 Å), SER494 (2.32, 2.89, 2.93 Å) |
| 17 | Didanosine | −5.2 | ARG403 (3.21, 3.25 Å), TYR453 (3.42, 3.51 Å), SER496 (2.29 Å), HIS505 (3.37 Å) |
| 18 | Famciclovir | −5 | TYR449 (2.88 Å), TYR453 (3.00 Å), TYR495 (2.52 Å), SER496 (2.96 Å), ARG498 (3.09, 3.22 Å) |
| 19 | Tenofovir | −5 | ARG403 (3.04, 3.30 Å), ASN417 (3.18 Å), TYR453 (3.19, 3.78, 4.17 Å), TYR495 (2.36, 3.43 Å), SER496 (2.70 Å) |
| 20 | Zalcitabine | −4.9 | SER496 (2.95, 3.73 Å), TYR501 (3.55 Å) |

^a^ Conventional hydrogen bonds, carbon-hydrogen bonds, and pi-donor hydrogen bonds (in Å) were listed.

**Table S6** Dataset of the studied anti-HIV agents, including PubChem ID, pharmacological indication, and group.

| No | Compound | PubChem ID | Indication | Group |
| --- | --- | --- | --- | --- |
| 1 | Simeprevir | 24873435 | Patients co-infected with HIV-1 and HCV Genotype 1 | Approved [1], [2] |
| 2 | Raltegravir | 54671008 | For the treatment of HIV-1 infection in conjunction with other antiretrovirals | Approved [3], [4] |
| 3 | Indinavir | 5362440 | For the treatment of HIV infection | Approved [5], [6] |
| 4 | Maraviroc | 3002977 | For the treatment of CCR5-tropic HIV-1 infection in adults and pediatric patients weighing at least 2kg | Approved, Investigational[7], [8] |
| 5 | Sofosbuvir | 45375808 | HIV/HCV Co-infected Patients | Approved [9], [10] |
| 6 | Nelfinavir | 64143 | For the treatment of HIV infection | Approved [11], [12] |
| 7 | Amprenavir | 65016 | For the treatment of HIV infection | Approved, Investigational [13], [14] |
| 8 | Delavirdine | 5625 | For the treatment of HIV-1 infection in combination with appropriate antiretroviral agents when therapy is warranted | Approved [15] |
| 9 | Darunavir | 213039 | For the treatment of human immunodeficiency virus (HIV) in children age 3 or above and adults with HIV-1 infection | Approved [16], [17] |
| 10 | Efavirenz | 64139 | For the treatment of HIV infection | Approved, Investigational [18], [19] |
| 11 | Elvitegravir | 5277135 | For the treatment of HIV infection | Approved [20], [21] |
| 12 | Nevirapine | 4463 | For use in combination with other antiretroviral drugs in the ongoing treatment of HIV-1 infection | Approved [22], [23] |
| 13 | Ritonavir | 392622 | Ritonavir is indicated in combination with other antiretroviral agents for the treatment of HIV-1 infection | Approved, Investigational [24], [25] |
| 14 | Sequinavir | 60787 | For the treatment of HIV-1 infection in patients 16 years of age and older | Approved, Investigational [26], [27] |
| 15 | Stavudine | 18283 | Used in the treatment of HIV infection. | Approved, Investigational |
| 16 | Lopinavir | 92727 | For the treatment of HIV-1 infection in adults and pediatric patients ≥14 days old | Approved [25], [28], [29] |
| 17 | Didanosine | 135398739 | For the treatment of HIV-1 infection in adults | Approved [30], [31] |
| 18 | Famciclovir | 3324 | For the treatment of HIV-1 infection, and manage herpes zoster. | Approved, Investigational [32] |
| 19 | Tenofovir | 464205 | A nucleotide analog indicated in the treatment of HIV infections | Experimental, Investigational [33] |
| 20 | Zalcitabine | 24066 | A dideoxynucleoside used to treat HIV | Approved, Investigational [19], [34] |

**Table S7** Compound names, docking scores (in kcal/mol), and binding features of twenty anti- bacterial compounds against Omicron variant of SARS-Cov-2

| **No** | **Compound** | **Docking Score (kcal/mol)** | **Binding Features** |
| --- | --- | --- | --- |
| 1 | Sarafloxacin | −7.2 | TYR453 (4.16 Å), SER494 (3.54 Å), SER496 (3.36 Å), TYR501 (2.69, 4.18, 4.20 Å) |
| 2 | Difloxacin | −7.0 | ARG403 (3.19 Å), TYR453 (4.18 Å), SER496 (3.55 Å), TYR501 (3.99 Å) |
| 3 | Trovafloxacin | −6.8 | ARG403 (3.28 Å), SER496 (3.26 Å), TYR501 (3.81, 4.06 Å) |
| 4 | Delafloxacin | −6.7 | TYR453 (3.95 Å), ARG493 (3.55 Å), SER494 (3.12 Å), SER496 (3.48 Å), |
| 5 | Orbifloxacin | −6.6 | ARG403 (3.50 Å), GLU406 (2.91 Å), TYR453 (2.87 Å), TYR495 (3.62 Å), TYR501 (3.52 Å) |
| 6 | Grepafloxacin | −6.5 | ARG403 (3.12, 3.27 Å), TYR453 (3.02 Å) |
| 7 | Levofloxacin | −6.3 | ARG403 (3.10, 3.12 Å), TYR453 (2.96 Å), SER496 (3.69 Å), TYR501 (3.53 Å) |
| 8 | Ofloxacin | −6.3 | ARG403 (3.40 Å), GLU406(2.71 Å), TYR453 (3.78, 3.27 Å), SER496 (3.43 Å), TYR501 (3.39 Å) |
| 9 | Lomefloxacin | −6.2 | ARG403 (2.89, 3.28 Å), TYR453 (3.06, 3.96 Å), SER496 (3.72 Å), TYR501 (3.41 Å) |
| 10 | Gatifloxacin | −6.1 | ASN417 (3.15 Å), TYR453 (4.06 Å), TYR495 (3.07, 3.63 Å), SER496 (3.31 Å), TYR501 (3.29 Å) |
| 11 | Moxifloxacin | −6 | ASN417 (2.97 Å), TYR453 (3.92 Å), SER496 (2.53 Å) |
| 12 | Temafloxacin | −6 | ARG403 (3.20 Å), SER496 (3.62 Å), TYR501 (3.93 Å) |
| 13 | Enoxacin | −6 | ARG403 (3.15 Å), TYR453 (3.06 Å), SER496 (3.58 Å), TYR501 (3.02 Å) |
| 14 | Ciprofloxacin | −5.9 | ARG403 (2.96 Å), ASN417 (3.06 Å), TYR453 (3.28, 3.85 Å), SER496 (3.20 Å), TYR501 (2.64 Å) |
| 15 | Norfloxacin | −5.9 | ARG403 (2.87 Å), GLU406 (2.70 Å), ASN417 (3.12 Å), SER496 (3.07 Å), TYR501 (2.77 Å) |
| 16 | Gemifloxacin | −5.9 | TYR453 (3.55, 3.94 Å), TYR495 (3.75 Å), TYR501 (3.01 Å) |
| 17 | Amifloxacin | −5.9 | ARG403 (3.10 Å), TYR453 (2.95 Å) |
| 18 | Fleroxacin | −5.8 | ARG403 (2.84, 3.32 Å), TYR453 (3.22 Å), SER496 (3.45 Å), TYR501 (3.43 Å) |
| 19 | Enrofloxacin | −5.7 | ARG403 (3.03 Å), TYR453 (2.86 Å), SER496 (3.53 Å) |
| 20 | Danofloxacin | −5.7 | TYR495 (2.27 Å), TYR500 (3.74 Å), TYR501 (3.55 Å), HIS505 (3.15 Å) |

^a^ Conventional hydrogen bonds, carbon-hydrogen bonds, and pi-donor hydrogen bonds (in Å) were listed.

**Table 8** Dataset of the studied anti-bacterial agents, including PubChem ID, pharmacological indication, and group.

| **No** | **Compound** | **PubChem ID** | **Indication** | **Group** |
| --- | --- | --- | --- | --- |
| 1 | Sarafloxacin | 56208 | A quinolone antibiotic drug, which was discontinued by its manufacturer, Abbott Laboratories, before receiving approval for use in the US or Canada. | Vet approved |
| 2 | Difloxacin | 56206 | A synthetic fluoroquinolone used in veterinary. As an antibacterial, it presents a broad bactericidal spectrum and its effects are dependent on its concentration | Vet approved |
| 3 | Trovafloxacin | 62959 | An antibiotic used to treat gonorrhea and chlamydia | Approved, Investigational, Withdrawn |
| 4 | Delafloxacin | 487101 | A fluoroquinolone antibiotic used to treat skin and skin structure infections | Approved, Investigational |
| 5 | Orbifloxacin | 60605 | A fluoroquinolone antibiotic. It is marketed by Schering-Plough Animal Health and approved for certain infections in dogs | Vet approved |
| 6 | Grepafloxacin | 72474 | A fluoroquinolone antibiotic used to treat various gram positive and gram-negative bacterial infections | Approved, Investigational, Withdrawn |
| 7 | Levofloxacin | 149096 | A fluoroquinolone antibiotic used to treat infections caused by susceptible bacteria of the upper respiratory tract, and skin | Approved, Investigational |
| 8 | Ofloxacin | 4583 | An antibacterial agent used for the treatment of bacterial infections in many parts of the body, including the respiratory tract, and kidney | Approved |
| 9 | Lomefloxacin | 3948 | A fluoroquinolone used to prevent and treat a wide variety of infections in the body | Approved, Investigational |
| 10 | Gatifloxacin | 5379 | A fourth-generation fluoroquinolone used to treat a wide variety of infections in the body | Approved, Investigational |
| 11 | Moxifloxacin | 152946 | A fluoroquinolone antibiotic used to treat various bacterial infections | Approved, Investigational |
| 12 | Temafloxacin | 60021 | An antibiotic agent belonging to the fluoroquinolone drug class | Not approved |
| 13 | Enoxacin | 3229 | A broad-spectrum 6-fluoronaphthyridinone antibacterial agent (fluoroquinolones) structurally related to nalidixic acid. | Approved, Investigational |
| 14 | Ciprofloxacin | 2764 | A second-generation fluoroquinolone used to treat various susceptible bacterial infections | Approved, Investigational |
| 15 | Norfloxacin | 4539 | A broad-spectrum fluoroquinolone antibiotic with variable activity against gram-positive and gram-negative bacteria | Approved |
| 16 | Gemifloxacin | 9571107 | A quinolone antibacterial agent used for the treatment of acute bacterial exacerbation of chronic bronchitis | Approved, Investigational |
| 17 | Amifloxacin | 55492 | A fluoroquinolone antibiotic, which is similar in its activity to ciprofloxacin | Not approved |
| 18 | Fleroxacin | 3357 | A broad-spectrum antimicrobial fluoroquinolone. It strongly inhibits the DNA-supercoiling activity of DNA gyrase. Fleroxacin is not an inhibitor of CYP1A2 | Experimental |
| 19 | Enrofloxacin | 71188 | An antibiotic agent from the fluoroquinolone family produced by the Bayer Corporation. | Vet approved |
| 20 | Danofloxacin | 71335 | An antibiotic agent from the family of the fluoroquinolones used in veterinary medicine | Experimental, Vet approved |


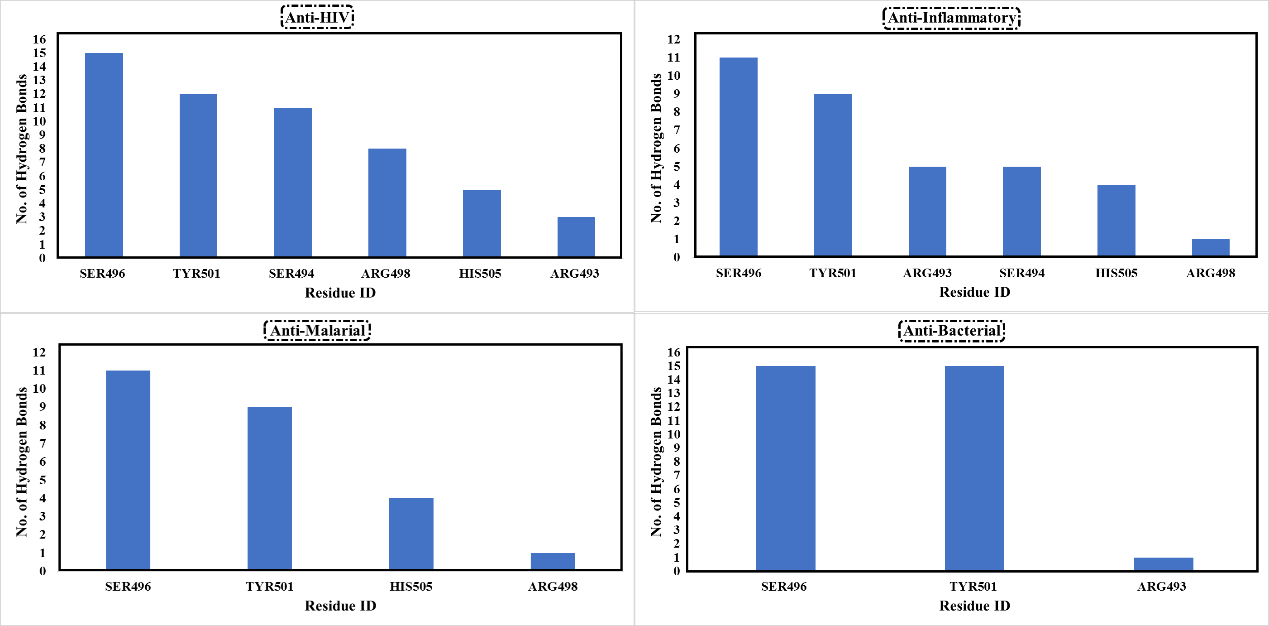


**Fig. S1** Number of hydrogen bonds formed between anti-HIV, anti-inflammatory, anti-malarial, and anti-bacterial agents with O-RBD.
